# A Quantitative Model of Chemotherapeutic Drug Sensitivity as a Function of P-Glycoprotein Expression

**DOI:** 10.3390/molecules30143014

**Published:** 2025-07-18

**Authors:** Cara M. Robertus, Nisha Kannan, David Putnam

**Affiliations:** 1 Meinig School of Biomedical Engineering, Cornell University, Ithaca, NY 14850, USA; cr546@cornell.edu (C.M.R.); nk489@cornell.edu (N.K.); 2 Smith School of Chemical and Biomolecular Engineering, Cornell University, Ithaca, NY 14850, USA

**Keywords:** P-glycoprotein, multidrug resistance, chemotherapeutic sensitivity, siRNA, drug-resistant cancer, efflux transporter

## Abstract

(1) Background: Overexpression of P-glycoprotein (P-gp) is one mediator of multidrug resistance in cancer. While many studies demonstrate the efficacy of modulating P-glycoprotein expression to increase drug response in cancer cells, the nature of the mathematical relationship between drug sensitivity and P-glycoprotein surface density is not yet characterized. (2) Methods: In this study, we employ siRNA to modulate P-gp expression in two model cell lines and evaluate their steady-state response to three common chemotherapeutics in vitro. Additionally, we model the kinetics of calcein-AM, a P-gp substrate, as a function of P-gp expression. (3) Results: For both cell lines, a robust linear relationship governs chemotherapeutic sensitivity as a function of P-gp expression, demonstrating that characterization of P-gp surface density is a strong indicator of drug response in drug-resistant cells. Furthermore, calcein accumulation and initial influx rate exhibit first-order kinetics with respect to P-gp density, further elucidating the nature of substrate interactions with P-gp-overexpressing cells. When transport kinetics are evaluated using a Michaelis–Menten model, V_max_ varies with P-gp density according to a first-order relationship. (4) Conclusions: These results establish the mathematical relationships between chemotherapeutic response and substrate influx as a function of P-gp expression and suggest that rational changes in P-gp expression could be used as a predictive measure of drug sensitivity in model cell lines.

## 1. Introduction

P-glycoprotein (P-gp) is an ATP-dependent transmembrane efflux transporter that binds and transports lipophilic molecules during passive diffusion across the cell membrane [1,2,3]. Expressed at boundary sites throughout the body, P-gp confers tissue protection from toxic compounds when expressed at natural barriers at endogenous levels [2,4]. However, P-gp overexpression is often observed in cancer cells and is associated with the development of multidrug resistance (MDR), an aggressive phenotype that renders patient tumors unresponsive to treatment and is correlated with reduced survival rates [5]. In some cases, elevated levels of P-gp expression are observed at the time of initial diagnosis, while in others it is detected only after administration of chemotherapy [6,7,8]. While early attempts to counteract the activity of P-gp in multidrug-resistant cancer cells relied on competitive inhibition, more recent work evaluates modulation of P-gp expression as a means to reverse drug resistance. Following early studies that established the efficacy of this strategy to re-sensitize drug-resistant cells, various drug delivery platforms have successfully co-delivered a P-gp modulator such as siRNA with a cytotoxic agent to eradicate drug-resistant cells in a single treatment [9,10,11,12,13,14]. Recent work has further demonstrated the viability of this strategy using next-generation strategies, such as monoclonal antibody targeting, immune cloaking, and even carrier-free systems [15,16,17]. However, while these studies affirm the utility of P-gp modulation as a therapeutic strategy, the impact of P-gp expression on outcome measures such as drug IC_50_ and substrate accumulation is difficult to contextualize. A quantitative understanding of how changes in P-gp expression correspond to measures of drug sensitivity is needed to define this relationship and enable prediction of cellular response to drugs. Furthermore, correlation of clinically relevant levels of P-gp expression with the response to chemotherapeutics is an important therapeutic goal, as it could inform dosing regimens on a patient-specific basis.

In this study, we use siRNA to quantitatively modulate P-gp expression in two distinct drug-resistant cell types: one that has been retrovirally transfected to constitutively express P-gp, and one that has been conditioned to express P-gp through prolonged drug exposure in culture. We correlate expression levels to IC_50_ parameters for three common chemotherapeutics to define the mathematical relationship between IC_50_ and quantitative P-gp expression. Furthermore, we demonstrate the impact of P-gp modulation on influx rates and accumulation of calcein-AM, a fluorescent substrate, to evaluate the kinetics of P-gp activity as a function of expression level at short timescales. The IC_50_ values for all drugs evaluated exhibit a linear dependence on P-gp expression that can be predictively modeled for individual cell lines. Furthermore, the kinetics of calcein-AM influx as a function of P-gp expression approach first-order behavior. Taken together, these data suggest that P-gp is the primary predictor of drug resistance in these MDR cells, and that changes in P-gp expression can be used to estimate corresponding changes in drug sensitivity in resistant cancer cells.

## 2. Results

### 2.1. Characterization of MDA435/LCC6 and MES-SA Cell Lines

To establish basic biological characteristics of cell lines used in this study, growth rates, membrane fluidity, and relative subcellular localization of P-glycoprotein expression were determined. Each of these factors can impact drug sensitivity, as membrane permeability, cell cycle characteristics, and P-glycoprotein expression influence cellular response to cytotoxins [18,19,20]. The doubling times of all cell lines under the specific growth conditions used in this study were comparable, with the only significant difference appearing between MES-SA cells and their drug-resistant counterpart, MES-SA/Dx5 (Figure S1A). The membrane fluidities of wild-type MDA435/LCC6 and MDA435/LCC6^MDR1^ cells were statistically equivalent, while MES-SA and MES-SA/Dx5 cells exhibited significantly higher and lower fluidities, respectively (Figure S1B). This trend could be due to selection of MES-Dx5 cells through prolonged exposure to a cytotoxin in culture, which may be associated with changes in membrane fluidity [21]. Finally, subcellular fractionation of membrane-associated and cytosolic proteins to determine the relative amounts of P-gp in each fraction revealed that P-gp is almost exclusively located in cell membranes for both cell lines (Figure S1C), suggesting that the contribution of cytosolic P-gp to drug efflux is minimal.

### 2.2. Modulation of P-Glycoprotein Expression Through siRNA Transfection

**Figure 1:**
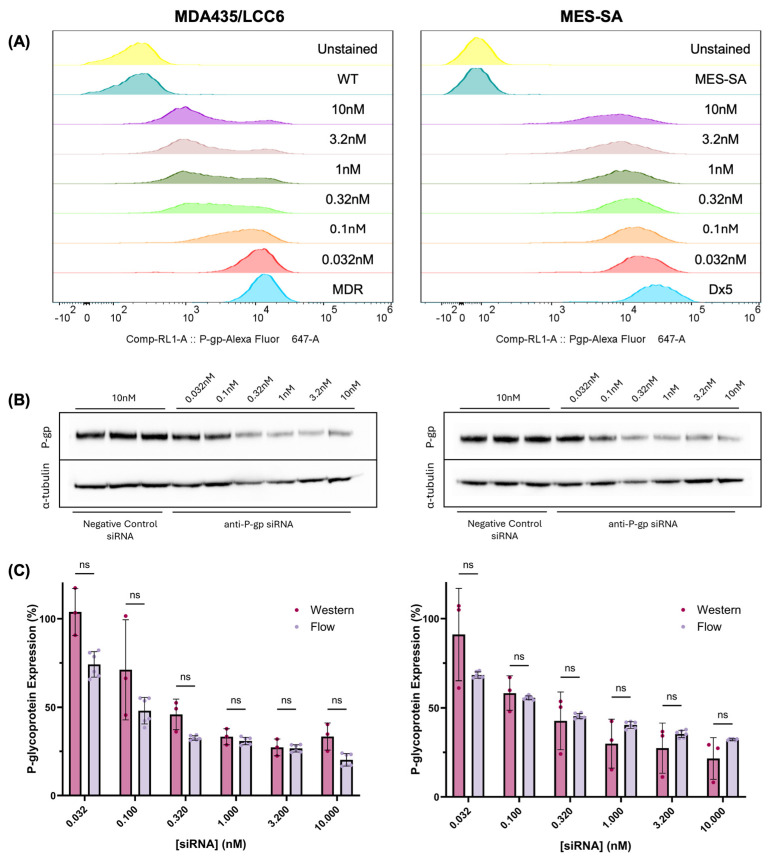
Modulation of P-glycoprotein expression in MDA435/LCC6 and MES-SA cells. (**A**) Flow cytometric population distributions of P-glycoprotein expression as measured by AlexaFluor 647-labeled anti-P-gp antibody binding. (**B**) Representative western blots of P-gp expression in total protein lysates of transfected cells. (**C**) Comparison between relative P-gp expression levels measured using western blot or flow cytometry. Significance measured using multiple Mann–Whitney tests. Error bars represent standard deviation; *n* = 3 for western blot; *n* = 6 for flow cytometry. “ns” represents “no significance,” indicating no statistical difference between groups.

Control of P-glycoprotein expression in individual cell lines was achieved using transfection with variable concentrations of siRNA ranging from 0.032 to 10 nM for all experiments. Control transfections with fluorescently-labeled TYE 563 positive control siRNA revealed high transfection efficiencies for both cell lines, demonstrating the validity of this approach (Figure S2A). Furthermore, RT-qPCR demonstrated successful recruitment of the RNA interference pathway, verifying the mechanism of P-gp modulation in this study (Figure S2B). Because no significant difference in P-gp mRNA fold change was observed across the three pre-designed siRNA sequences evaluated, the first sequence (hs.Ri.ABCB1.13.1) was arbitrarily selected for use in all subsequent transfections. In this study, siRNA was being used simply as a tool to rationally modulate P-gp expression; therefore, the specific siRNA sequence selected was considered to be of little consequence. A transfection protocol consisting of 24 h of siRNA exposure followed by an additional incubation period of 72 h for a total post-transfection duration of four days was determined to yield maximum P-gp knockdown in MDA435/LCC6^MDR1^ cells; therefore, this time point was selected for use in all subsequent transfections (Figure S3). Because substantial knockdown of P-gp was also observed using an identical transfection protocol in MES-SA/Dx5 cells, the same procedure was used for both to maintain consistency between the cell lines. Flow cytometry analysis revealed differences in the population distributions of P-glycoprotein expression in transfected cells (Figure 1A). While MDA435/LCC6 populations exhibited a bimodal distribution consisting of a small number of resistant cells coupled with an increasing number of sensitive cells, MES-SA populations maintained a Gaussian-like distribution. In both lines, a progressive shift towards the drug-sensitive phenotype is observed with increasing concentrations of siRNA. This population-level change in P-gp expression is reflected in total protein lysates analyzed using western blot (Figure 1B). Importantly, there is no significant difference between relative P-gp expression measured using western blot or flow cytometry, validating the use of surface staining as an approach to measure P-gp content. In the graphs that follow, “P-glycoprotein expression” reflects values measured using flow cytometry, as these measurements were more precise relative to western blot. Numerical surface expression values for all cell populations are shown in Table S1. All western blots and flow cytometry gating schemes can be found in Figures S4–S9.

### 2.3. Determination of Sensitivity of Transfected Cells to Chemotherapeutics

**Figure 2:**
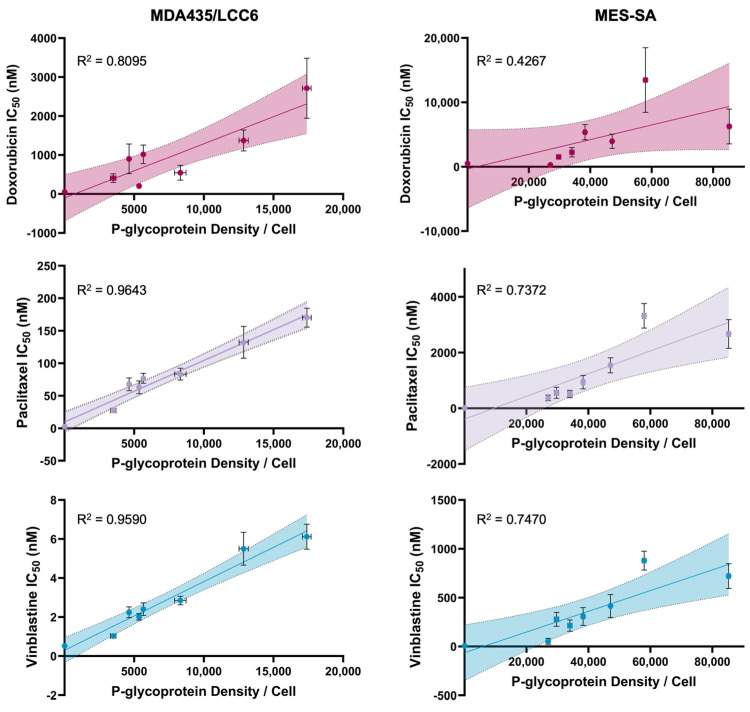
Relationship between IC_50_ and P-glycoprotein expression in MDA435/LCC6 and MES-SA cells. Linear plots of measured IC_50_ values for doxorubicin, paclitaxel, and vinblastine versus P-glycoprotein expression in cells transfected with varying concentrations of anti-P-gp siRNA ranging from 0.032 to 10 nM. Vertical error bars represent the standard error of the IC_50_ value derived from nonlinear regression of cell viability data, *n* = 6. Horizontal error bars represent the standard error of P-glycoprotein expression measured using flow cytometry, *n* = 6. Shaded areas represent the 95% confidence interval of the best-fit line.

To characterize the relationship between drug sensitivity and P-glycoprotein expression in both constitutively transfected and conditioned drug-resistant cells, cell viability assays were performed using three common chemotherapeutics: doxorubicin, paclitaxel, and vinblastine. While structurally and mechanistically diverse, each of these compounds is a substrate for P-gp, rendering them suitable representative candidates for this study. For each substrate, IC_50_ values were determined for wild-type and drug-resistant cells transfected with negative control siRNA, as well as resistant cells transfected with varying concentrations of anti-P-gp siRNA. These data were plotted as a function of quantitative P-glycoprotein expression as determined by flow cytometry. As shown in Figure 2, these relationships exhibit strong linear correlations, indicating that drug sensitivity is directly proportional to P-gp content in the cell membrane under steady-state conditions. Model equations for each pairing of cell type and substrate were derived using simple linear regression, which was determined to appropriately represent the data based on the strength of the correlation coefficient relative to alternate models. Furthermore, the observation of this relationship in both MDA435/LCC6 and MES-SA cells, despite the biological differences between the cell lines, suggests that P-gp content is the primary predictor of drug sensitivity in this system. Model parameters for each linear relationship are shown in Table 1. All IC_50_ curves can be found in Figures S10 and S11.

### 2.4. Characterization of Cell Interactions with Calcein-AM

**Figure 3:**
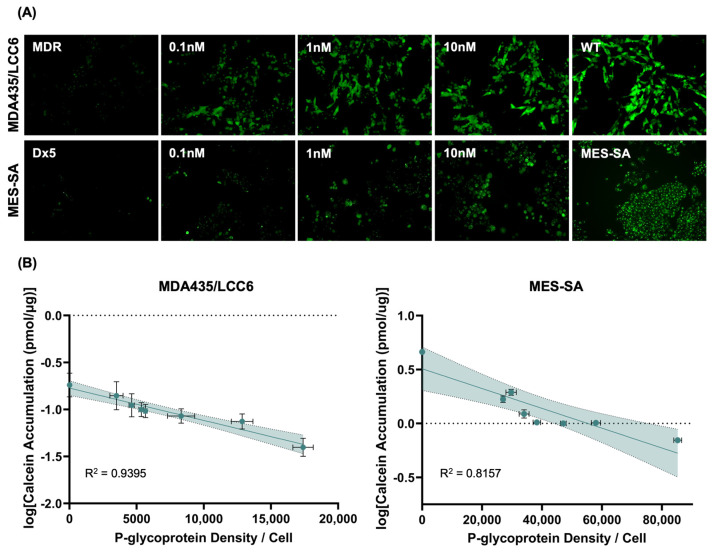
Calcein-AM accumulation in MDA435/LCC6 and MES-SA cells. (**A**) Representative fluorescent microscopy images of calcein-AM accumulation and subsequent cleavage in cells transfected with varying concentrations of anti-P-gp siRNA ranging from 0.032 to 10 nM. (**B**) Semi-log plots of calcein concentration measured in cell lysates using fluorescent spectrophotometry after steady-state accumulation versus P-glycoprotein expression. Data normalized to total protein as a measure of cell density. Vertical error bars represent standard deviation of the logarithm of calcein concentration, *n* = 6. Horizontal error bars represent standard deviation of P-gp expression measured using flow cytometry, *n* = 6. Shaded areas represent the 95% confidence interval of the best-fit line.

Because drug sensitivity studies were performed over long time scales (72 h of drug exposure) and rely on cell death as a proxy for substrate accumulation, additional studies to directly measure the interaction of P-gp-expressing cells with calcein-AM over short time scales were undertaken. In its native state, calcein-AM is not fluorescent; however, upon internalization by cells, it is cleaved by intracellular enzymes into its fluorescent form, rendering it an ideal test substrate for P-glycoprotein efflux studies. Importantly, because calcein fluoresces only upon internalization by the cell following P-gp bypass, this approach enables direct measurement of substrate influx, demonstrating inverse trends relative to approaches that directly measure P-gp efflux. Microscopy studies revealed a clear correlation between P-gp content and calcein fluorescence after 30 min of exposure in both MDA435/LCC6 and MES-SA cells (Figure 3A). Similarly, cell lysates evaluated after 60 min of exposure exhibited a consistent trend, demonstrating a decrease in calcein fluorescence with increasing expression of P-glycoprotein at the cell membrane (Figure 3B). Interestingly, calcein accumulation did not exhibit a linear relationship with P-gp expression (unlike the IC_50_ studies), but rather a first-order relationship, as demonstrated by the linear correlation between the logarithm of calcein signal versus P-gp content. This finding suggests that P-gp substrate interactions over short time scales may be governed by different relationships than those observed at steady state.

**Figure 4:**
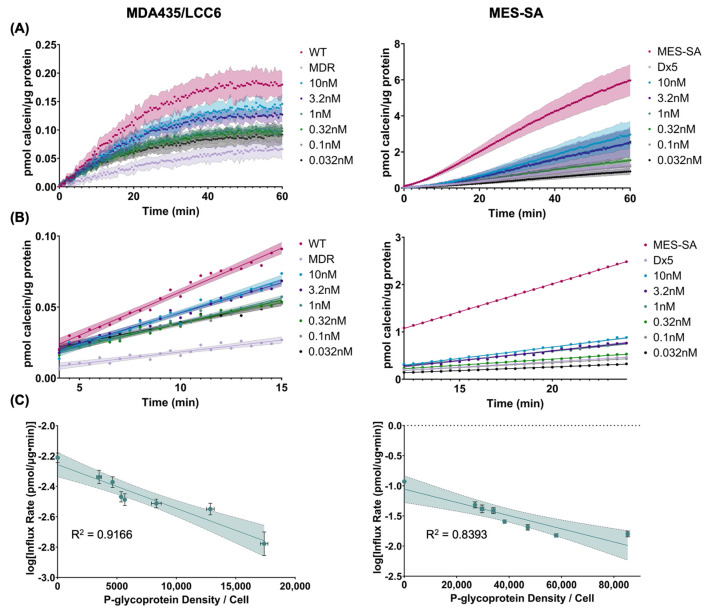
Kinetics of calcein accumulation in MDA435/LCC6 and MES-SA cells. (**A**) Kinetic curves of calcein accumulation over time in cells transfected with varying concentrations of anti-P-gp siRNA ranging from 0.032 to 10 nM. Data are normalized to total protein as a measure of cell density. Shaded areas represent standard deviation of calcein fluorescence, *n* = 6. Legend values represent siRNA concentrations used to modulate P-gp expression. (**B**) Linear portions of plots in (**A**) used to estimate calcein influx rates. For clarity, error bars from (**A**) have been removed, and shaded areas represent the 95% confidence interval of the best-fit line using simple linear regression. Legend values represent siRNA concentrations used to modulate P-gp expression. (**C**) Semi-log plots of the influx rate of calcein as a function of P-glycoprotein expression. Vertical error bars represent the standard error of the slope of the best-fit lines estimated from the plots in (**B**). Horizontal error bars represent the standard error of the mean of P-glycoprotein expression measured using flow cytometry, *n* = 6. Shaded areas represent the 95% confidence interval of the best-fit line.

To evaluate the kinetics of calcein accumulation in cells with variable P-gp expression, calcein fluorescence was measured as a function of time over a period of 1 h (Figure 4A). Rates of calcein influx were estimated by performing simple linear regression on the linear subsections of the curves prior to attainment of saturation (Figure 4B). The correlation between calcein influx rate and P-glycoprotein expression can be described by a first-order relationship, as demonstrated by linear slopes of plots of the logarithm of calcein influx rate vs. P-glycoprotein content (Figure 4C). These data correspond with the trends observed for quantitative calcein accumulation described in Figure 3B, strengthening the conclusion that the linear relationship observed between substrate IC_50_ and P-gp expression does not govern interactions over short time scales. Notably, the first-order correlation between influx rate and P-gp expression is stronger for MDA435/LCC6 cells than for MES-SA cells, suggesting that the influx rate of calcein over short time scales may be influenced by factors beyond P-gp. Model parameters of best-fit lines correlating calcein accumulation and influx rates to P-glycoprotein expression are shown in Table 2.

### 2.5. Determination of Michaelis–Menten Characteristics of MES-SA Cells

**Figure 5:**
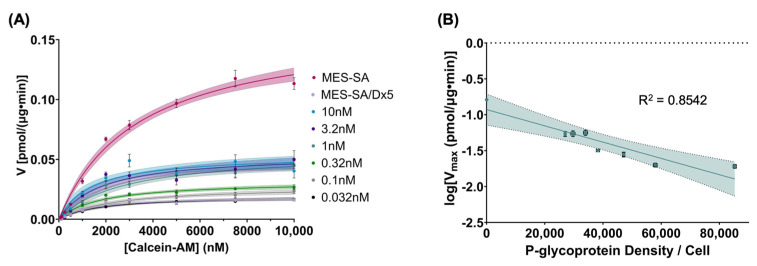
Michaelis–Menten behavior of MES-SA cells treated with calcein-AM. (**A**) Michaelis–Menten curves for each transfected population of MES-SA cells. Shaded areas represent the 95% confidence interval of the best-fit line using a nonlinear regression model. Error bars represent the standard error of the mean of initial influx rates. Legend values represent siRNA concentrations used to modulate P-gp expression. (**B**) Semi-log plot of V_max_ as a function of P-glycoprotein surface expression. Vertical error bars represent the standard error of the V_max_ values calculated from (**A**), *n* = 6. Horizontal error bars represent the standard error of the mean of P-glycoprotein expression measured using flow cytometry, *n* = 6. Shaded area represents the 95% confidence interval of the best-fit line.

To further evaluate the kinetic behavior of the MES-SA cell line, kinetic studies were performed over a range of calcein-AM concentrations to calculate the maximum influx rate for each cell population (V_max_) and the substrate concentration at which half of the maximum influx rate was achieved (K_m_). Furthermore, the relationship between these parameters and P-glycoprotein surface density was evaluated. As shown in Figure 5A, the initial influx rate of calcein demonstrated saturable behavior as a function of initial substrate concentration, exhibiting the characteristic shape expected for a Michaelis–Menten curve and enabling V_max_ and K_m_ values to be estimated for each population. When evaluated as a function of P-glycoprotein surface density, V_max_ values exhibited a first-order correlation, in line with the kinetic behavior observed in the previous experiment (slope = −1.133 × 10^−5^ ± 1.911 × 10^−6^; y-intercept = −0.9278 ± 0.08831) (Figure 5B). Interestingly, the correlation between K_m_ and P-glycoprotein surface density was weak for both linear and log-transformed data (Figure S12). This result suggests that additional factors may govern the Michaelis–Menten parameters of this system.

## 3. Discussion

The objective of this study was to determine the relationship between drug sensitivity and P-glycoprotein expression using two biologically distinct cell lines as model systems. Both steady-state behavior and short-term kinetics were explored using chemotherapeutics and calcein-AM as substrates, respectively. While IC_50_ values were found to correlate with P-glycoprotein expression via a strong linear relationship in both MDA435/LCC6 and MES-SA cells, calcein accumulation and influx rate were more closely modeled by first-order kinetics. Furthermore, the influx rates observed in MES-SA cells exhibited distinct behavior compared to MDA435/LCC6^MDR1^ cells, suggesting that biological variables may play a more substantial role in governing short-term substrate interactions.

MDA435/LCC6^MDR1^ cells were retrovirally transfected to express P-glycoprotein, while MES-SA/Dx5 cells were drug-selected following prolonged exposure to doxorubicin in culture. Importantly, cell lines used to investigate drug resistance broadly fall into these two categories, underscoring the relevance of this work to future in vitro studies. While growth rates and membrane fluidities were similar across all cell lines and phenotypes, several important differences were observed (Figure S1). Notably, the significantly different growth rates and membrane fluidities of MES-SA and MES-SA/Dx5 cells may contribute to unexpected trends observed when data from both cell lines and transfected populations of MES-SA/Dx5 cells are taken together.

In this work, anti-P-gp siRNA was successfully used to modulate P-glycoprotein expression in MDA435/LCC6^MDR1^ and MES-SA/Dx5 cells, as shown in Figure 1. Notably, distinct population distribution trends appear in each cell type; MDA435/LCC6^MDR1^ cells exhibit bimodal distributions as a growing subpopulation shifts toward the drug-sensitive phenotype, while a small population of resistant cells persists. In contrast, MES-SA/Dx5 cells exhibit a Gaussian-like population distribution that progressively drifts toward the drug-sensitive phenotype. This difference may be due to the significant variability of P-glycoprotein half-life among cell lines, with reported values ranging from 15–72 h [21,22,23,24]. A longer half-life would result in a slower return to the drug-resistant phenotype, both at the single-cell and population level.

To quantify P-glycoprotein expression in this study, flow cytometry experiments were performed using saturating concentrations of anti-P-glycoprotein Alexa Fluor 647 antibody, and quantification of receptor density was performed assuming 1:1 binding between antibody and P-gp. To validate this assumption, western blot quantification was also performed to determine if any significant deviation was observed from values determined using flow cytometry. Importantly, western blot quantifies total protein content, including P-glycoprotein located in intracellular compartments. The secretory pathway of P-gp includes the endoplasmic reticulum, the Golgi body, the early endosome, and the lysosome, but only cell membrane-associated P-glycoprotein contributes significantly to drug resistance [25,26,27,28]. For the cell lines evaluated in this study, a negligible amount of P-gp was found in cytosolic protein fractions, suggesting that almost all P-gp is membrane-associated (Figure S1C). Therefore, relative P-gp expression measured using western blot and flow cytometry can be reasonably compared. The statistical equivalence between values measured using both methods suggests that the assumptions underlying the flow cytometry quantification approach are valid, legitimizing its use to derive values employed in the models reported throughout this work (Figure 1C).

To determine the effect of modulating P-glycoprotein expression on drug sensitivity in vitro, cell viability assays were performed to calculate IC_50_ values for three common chemotherapeutics in cells treated with both negative control siRNA and a range of anti-P-gp siRNA concentrations. As shown in Figure 2, these data demonstrate a strong linear correlation for both cell lines in all drugs evaluated, suggesting that P-glycoprotein expression is the primary driving force of cellular response to substrates in this in vitro model and may be used to predict drug resistance within this defined biological system. These results align with a previous study in which murine NIH 3T3 cells retrovirally transfected to express variable amounts of P-glycoprotein demonstrated linear correlations between both vinblastine and colchicine resistance and relative P-gp density [29]. Importantly, this work extends these findings to a more clinically relevant drug-selected line, which is often characterized by additional biochemical changes relative to its parent lines [30,31]. However, it is crucial to note that multidrug resistance is a complex and multifaceted phenotype that is mediated by numerous additional mechanisms, including mutations in drug targets, epigenetic changes, enhanced DNA damage repair, and changes in cell signaling pathways and the tumor microenvironment [5]. Importantly, several of these factors are likely to play a more significant role in vivo than in vitro, underscoring the need for modeling with additional cell lines and in more complex biological systems to fully establish the relationship between P-gp expression and drug response. Nonetheless, these preliminary results suggest that P-glycoprotein expression may serve as a predictor of drug resistance in both retrovirally transfected and drug-selected cell lines in vitro. By modulating P-gp expression within both lines using siRNA rather than increasing cytotoxic selective pressure, further induction of biological changes in response to drug treatment was minimized.

Notably, the best-fit parameters of the linear models differ as a function of both substrate and cell line; therefore, a unifying model in which only P-gp expression can be used to directly predict IC_50_ for any system cannot be derived from these data alone, limiting their predictive power. Indeed, the degree of resistance of a given cell type to a substrate can be described by the following equation:

(1) R=(P+k)/P

in which *R* represents the degree of resistance, *P* represents the permeability of the cell to the substrate, and *k* represents the rate constant of P-gp efflux [32]. Because *P* depends on both the specific biological properties of the cell type of interest and the physical and chemical properties of the substrate, *R* cannot be directly predicted from P-gp expression without first establishing the boundary conditions of the biological system. This is demonstrated in Figures S10 and S11, in which IC_50_ values of the drug-sensitive parent lines MDA435/LCC6 and MES-SA, both of which express undetectable levels of P-glycoprotein, are significantly different across all substrates evaluated. However, this study establishes a general equation that can be used to predict drug sensitivity within a given biological system as follows:

(2) $${IC}_{50}=\left[\frac{Pgp-{Pgp}^{S}}{{Pgp}^{R}-{Pgp}^{S}}\times ({IC}_{50}^{R}-{IC}_{50}^{S})\right]+{IC}_{50}^{S}$$

wherein *R* parameters indicate measured values for the resistant phenotype and *S* parameters indicate measured values for the drug-sensitive phenotype. This model accounts for biological variability in *P* by contextualizing changes in P-gp expression and drug sensitivity relative to the properties of the parent cell line. By evaluating the relationship between P-gp expression and drug sensitivity in two distinct tissue types representing both retrovirally transfected and drug-selected cells, this study lays important groundwork for further elucidating the role of P-gp in multidrug resistance in vitro. To complement this work, additional cell lines and chemotherapeutic substrates must be screened to fully establish the predictive power of this model for in vitro applications.

While these results describe the relationship between P-gp expression and drug sensitivity in vitro, they may have implications for clinical drug resistance as well. Previous work elucidated a strong correlation between P-gp expression and response to both paclitaxel and doxorubicin in 185 clinical samples of breast carcinoma, suggesting that in vitro findings may be descriptive of clinical phenomena [33]. Furthermore, expression levels of P-glycoprotein correlated with both in vitro sensitivity to daunorubicin and clinical outcomes in 36 patients with adult acute leukemia, demonstrating that this behavior is observed across drug-resistant cancers of various types [34]. Though these initial findings are promising, it is critical to extend this work to an in vivo system to broaden the predictive scope and clinical relevance of the model described herein. Importantly, 2D culture systems possess inherent drawbacks, including changes in cell morphology, altered cell signaling, and a lack of the heterogeneous cell types that comprise the tumor microenvironment [35]. These factors may result in distinct behavior relative to 3D culture platforms or in vivo models, underscoring the importance of additional model systems, such as tumor spheroids or xenograft studies, to validate these findings. Another approach could leverage existing clinical data, such as those contained in the Cancer Cell Line Encyclopedia, to construct a data-driven model to correlate patient response to various chemotherapeutics as a function of P-gp expression with our in vitro findings [36]. Such an approach could pave the way to evaluation of clinical samples, such as patient biopsies, to predict drug response and develop appropriate treatment regimes, but significant validation must be done to determine whether tumor P-gp expression levels can be used as a reliable indicator of chemotherapeutic susceptibility.

To further describe the relationship between P-glycoprotein expression and substrate interactions, we evaluated the kinetics of calcein-AM transport and accumulation in transfected MDA435/LCC6 and MES-SA cells. Calcein-AM is uniquely suited as a fluorescent probe to study P-glycoprotein transport, as it is fluorescent only upon cellular internalization and subsequent cleavage by intracellular enzymes [37]. Thus, it enables direct measurement of P-gp activity, as any measured fluorescence is inversely proportional to P-gp efflux. As shown in Figure 3, calcein accumulation was inversely correlated with increasing P-glycoprotein expression for both cell lines evaluated. These results align with a previous study in which calcein accumulation was shown to decrease with P-gp expression in various drug-resistant lines of both human and murine origin [38]. The data presented herein indicate a first-order relationship between calcein accumulation and P-gp expression, in contrast to the linear relationships seen in IC_50_ studies. This may suggest that longer timescales are required to achieve steady-state in these cell lines. While saturation behavior is observed, as shown in Figure 4A, trapping of substrates in the cell membrane during transport may contribute to extended periods of slow increases in intracellular concentration, prolonging the time to reach equilibrium [39]. A similar trend is observed when influx rates are derived from the kinetic curves and plotted against P-gp expression, as shown in Figure 4B,C.

Total substrate transport in cells expressing P-gp can be described by the following equation:

(3) $$v=k∆\left[S\right]-\frac{V_{max}[S]}{K_{m}+[S]}$$

in which *k* represents the passive permeation coefficient of the substrate, Δ*S* represents the concentration gradient of substrate across the extracellular and intracellular compartments, V_max_ represents the maximum efflux rate at saturation, *S* represents the substrate concentration, and K_m_ represents the substrate concentration at which half of the P-gp binding sites are saturated [40]. The first term describes passive transport of substrate across the cell membrane, while the second term describes the Michaelis–Menten behavior of P-gp transport kinetics. In this system, we assumed that passive transport of calcein-AM is constant within a given cell line; thus, we chose to further investigate the Michaelis–Menten behavior of the MES-SA cell line over a broad range of calcein-AM concentrations to evaluate the relationship between V_max_ and K_m_ and P-glycoprotein density (Figure 5). While V_max_ exhibited a first-order relationship with P-gp expression, in line with our previous kinetics experiments, there was a notably weaker correlation between K_m_ and P-gp surface expression (Figure S12). This may suggest that additional factors contribute to the kinetic behavior of this system, or that alternate models could be used to more fully describe the system. Specifically, passive transport may be variable as a function of membrane fluidity, which could be modulated by P-gp density. Notably, previous work evaluating the apical to basal transport of Caco-2 cells exhibiting variable levels of P-gp expression found a linear correlation between both K_m(app)_ and V_max_ and P-gp content [41]. Our results may indicate that the kinetic behavior of P-gp may vary as a function of system architecture, as we evaluated intracellular accumulation rather than apical to basal transport. While further studies should be undertaken to shed more light on the kinetic behavior of drug-resistant cells as a function of P-gp expression, the findings reported herein demonstrate that P-glycoprotein expression is a strong predictor of both drug sensitivity and substrate transport in vitro.

## 4. Materials and Methods

### 4.1. Materials

Modified improved minimum essential medium, McCoy’s 5a medium, penicillin/streptomycin, heat-inactivated fetal bovine serum, 0.25% trypsin-EDTA, Pierce BCA Protein Assay Kit, Mem-PER Plus Membrane Protein Extraction Kit, Pierce protease inhibitor mini tablets (EDTA-free), Lipofectamine RNAiMAX, NuPage 10% Bis-Tris gels, NuPAGE MOPS buffer, NuPAGE sample buffer, NuPAGE sample reducing agent, eBioscience™ Fixable Viability Dye eFluor™ 780, SuperScript III First-Strand Synthesis Kit, 16% *w*/*v* aqueous solution paraformaldehyde, doxorubicin hydrochloride, vinblastine sulfate, and eBioscience calcein-AM viability dye were obtained from Thermo Fisher Scientific (Waltham, MA, USA). Cell culture-grade sterile phosphate buffered saline, RIPA lysis buffer, trypan blue, and ultrapure-grade calcein were purchased from VWR (Radnor, PA, USA). Monoclonal anti-P-glycoprotein antibody produced in mouse, monoclonal anti-α-tubulin antibody produced in mouse, and anti-mouse IgG horseradish peroxidase antibody produced in rabbit were obtained from Sigma Aldrich (St. Louis, MO, USA). Nonfat dry milk, PVDF membrane, Clarity Western ECL substrate, and Precision Plus Protein Dual Color Standard were purchased from Bio-Rad (Hercules, CA, USA). Anti-P-glycoprotein Dicer-substrate siRNA (hs.Ri.ABCB1.13.1, hs.Ri.ABCB1.13.2, and hs.Ri.ABCB1.13.1.3), negative control Dicer-substrate siRNA, TYE 563-labeled transfection control Dicer-substrate siRNA, positive control anti-HPRT-S1 Dicer-substrate siRNA, and PCR primers were obtained from Integrated DNA Technologies (Coralville, IA, USA). All siRNA sequences were included in the predesigned TriFECTa^®^ DsiRNA kit against abcb1. Monoclonal Alexa Fluor 647 anti-P-glycoprotein antibody produced in mouse and membrane fluidity kit were purchased from Abcam (Cambridge, UK). The RNase-Free DNAse set and the RNeasy Mini Kit were obtained from Qiagen (Germantown, MD, USA). CellTiter 96^®^ AQueous One Solution Cell Proliferation Assay (MTS) was purchased from Promega (Madison, WI, USA). Luna Universal qPCR Master Mix was obtained from New England Biolabs (Ipswich, MA, USA). Quantum Alexa Fluor 647 MESF beads were purchased from Bangs Laboratories. Paclitaxel was a generous gift from Samyang Biopharma (Seongnam, Republic of Korea).

### 4.2. Cell Lines

Wild-type MDA435/LCC6 and drug-resistant MDA435/LCC6^MDR1^ human melanoma cells were a generous gift from Dr. Robert Clarke at Georgetown University. MDA435/LCC6^MDR1^ cells were retrovirally transfected to constitutively express P-glycoprotein [42,43]. Wild-type MES-SA and drug-resistant MES-SA/Dx5 human uterine sarcoma cells were a generous gift from Dr. Tamara Minko at Rutgers University. MES-SA/Dx5 cells were conditioned to express P-glycoprotein through prolonged exposure to doxorubicin [44].

### 4.3. Cell Culture

All cells were maintained in a humidified atmosphere at 37 °C and 5% CO_2_. MDA435/LCC6 and MDA435/LCC6^MDR1^ cells were grown in modified improved minimum essential medium (IMEM), and MES-SA and MES-SA/Dx5 cells were maintained in McCoy’s 5a growth medium. All growth media was supplemented with 10% fetal bovine serum and 1% penicillin/streptomycin unless noted otherwise. To maintain the drug-resistant phenotype in MES-SA/Dx5 cells, growth medium supplemented with 1 µM doxorubicin was added for a duration of 48 h every third passage. Cells were passaged at least once per week, and maintenance of the drug-resistant phenotype in MES-SA/Dx5 cells was periodically verified using flow cytometry.

### 4.4. Characterization of Cell Lines

To compare key biological characteristics of cell lines, several analyses were performed. First, the growth rate of each cell line was determined by seeding 18,000 cells per well of a 6-well plate in triplicate in appropriate growth medium. Every 24 h for 10 days, cells were washed with PBS, lifted using 0.25% trypsin EDTA, and incubated for approximately 5 min at 37 °C and 5% CO_2_. Following incubation, trypsin was neutralized using an equal volume of culture medium, and cells were pelleted at 1000 rpm for 5 min at 4 °C. The supernatant was discarded, and cells were resuspended in culture medium, stained with trypan blue, and counted using a Nexcelom Cellometer Auto 2000 Cell Viability Counter (Nexcelom, Lawrence, MA, USA). Total cell counts were plotted against time, and growth rates were determined using an exponential growth model in GraphPad Prism 10.2.0.

To determine the membrane fluidity of each cell line, cells were seeded in V-bottom plates at a density of 100,000 cells per well and washed with ice-cold PBS. Following centrifugation at 1500 rpm for 5 min at 4 °C, cells were incubated on ice with gentle agitation in a 50 µL solution containing 10 µM pyrene decanoic acid (PDA) and 0.08% Pluronic F-127 (membrane fluidity kit, Abcam, Cambridge, UK) for 1 h. Non-incorporated PDA was removed by two PBS washes and centrifugation, and cells were resuspended in 100 µL PBS. Fluorescence measurements were taken at 350/400 and 350/470 nm excitation/emission on a SpectraMax Gemini EM fluorescent plate reader (Molecular Devices, San Jose, CA, USA). Membrane fluidity was determined by measuring the ratio of incorporated (470 nm) to non-incorporated (400 nm) PDA.

To evaluate the relative content of membrane-associated and cytosolic P-glycoprotein, subcellular fractionation was performed using the Mem-PER Plus Membrane Protein Extraction Kit according to the manufacturer’s instructions. Briefly, 5 × 10^6^ cells were washed with 3 mL of cell wash solution and centrifuged at 300× *g* for 5 min at 4 °C. The supernatant was discarded, and cells were washed in 1.5 mL of cell wash solution. Following centrifugation, cells were resuspended in 0.75 mL of permeabilization buffer supplemented with protease inhibitors and vortexed briefly. After incubation for 10 min at 4 °C with constant mixing, cells were centrifuged at 16,000× *g* for 15 min at 4 °C. Supernatant containing cytosolic proteins was transferred to a new tube. To the remaining cell pellet, 0.5 mL of solubilization buffer supplemented with protease inhibitors was added, and cells were briefly vortexed. Cells were incubated at 4 °C with constant mixing for 30 min, followed by centrifugation at 16,000× *g* at 4 °C. Supernatant containing membrane-associated proteins was transferred to a new tube. Protein content of both cytosolic and membrane-associated fractions were determined using the Pierce BCA assay kit, and relative distribution of P-gp was determined using western blot as described below.

### 4.5. Modulation of P-Glycoprotein Expression Through siRNA Transfection

To modulate P-glycoprotein expression, cells were transfected with varying concentrations of anti-P-gp siRNA. All siRNA sequences were predesigned and were obtained from the TriFECTa^®^ DsiRNA kit targeted against abcb1. Cells were seeded at appropriate densities in 6-well plates for western blot and flow cytometry, 24-well plates for transfection control and calcein microscopy studies, and 96-well plates for drug sensitivity and calcein kinetics studies. All seeding densities are shown in Table S2. Following seeding, cells were incubated overnight to facilitate attachment. Prior to transfection, cells were washed with PBS and bathed in culture media supplemented with 10% fetal bovine serum without antibiotic. To transfect cells, suspensions of Lipofectamine RNAiMAX were prepared according to the manufacturer’s protocol. Dilution series (0.032 nM, 0.1 nM, 0.32 nM, 1 nM, 3.2 nM, and 10 nM) of anti-P-gp siRNA and a TYE 563-labeled fluorescent control siRNA were prepared in supplement-free culture medium. Three pre-designed P-gp targeted sequences were evaluated, and the sequence selected for use was 5′-r(AACUUGAAAGGUCAACAAAAAUTT) (sense) and 5′-r(AAAUUUUUGUUGUACCUUUCAAGUUCU) (antisense). As a negative control, 10 nM non-targeting siRNA was used for all studies. The transfection complex was prepared by gently pipette mixing the Lipofectamine and siRNA solutions and incubating at room temperature for 10 min. Appropriate siRNA/Lipofectamine complexes were then added to each well and incubated for 24 h at 37 °C and 5% CO_2_. Following incubation, cells were either rinsed with PBS and treated with appropriate drug treatments as described below or washed with PBS and treated with appropriate growth medium supplemented with 10% fetal bovine serum. To verify transfection, cells treated with the fluorescently labeled transfection TYE 563 control siRNA were imaged using a BZ-X810 fluorescent microscope (Keyence, Itasca, IL, USA) equipped with a TRITC filter.

### 4.6. Determination of P-Glycoprotein mRNA Expression Using Real-Time Quantitative PCR

To confirm recruitment of the RNA interference pathway following transfection with siRNA, a control experiment was performed in MDA435/LCC6 cells. Briefly, cells were transfected in triplicate with 20 nM of a positive control siRNA designed to modulate expression of the housekeeping gene HPRT and three pre-designed sequences targeted against P-gp. Following 24 h of transfection, RNA was extracted using the Qiagen RNeasy Mini Kit according to the manufacturer’s protocol. DNase digestion was performed on the extraction column using the RNase-Free DNase Set according to the manufacturer’s instructions. RNA concentration was measured using a NanoDrop One UV-Vis Spectrophotometer (ThermoFisher Scientific, USA). cDNA was then produced using the SuperScript III First-Strand Synthesis Kit. Briefly, 8 µL of RNA was combined with 1 µL of Oligo(dT)_20_ and 1 µL of 10 mM dNTP mix. Samples were incubated at 65 °C for 5 min and chilled on ice. To each tube, a mixture of 2 µL 10x RT buffer, 4 µL 25 mM MgCl_2_, 2 µL 0.1 M DTT, 1 µL RNase OUT, and 1 µL of SuperScript III RT was added, and samples were incubated at 50 °C for 50 min. Reactions were then terminated at 85 °C for 5 min and chilled on ice. Finally, 1 µL of RNaseH was added to each tube, and samples were incubated for 20 min at 37 °C. cDNA samples were used to directly proceed to qPCR.

qPCR samples were prepared by combining a volume of cDNA produced from 100 ng equivalent RNA with 0.5 µL of 10 µM forward primer, 0.5 µL of 10 µM reverse primer, and 10 µL of Luna Universal qPCR Master Mix. Samples were prepared to evaluate expression of both target genes (HPRT and P-gp) as well as β-actin as an internal reference. The following primers were used: β-actin forward: 5′-GTGGGGCGCCCCAGGCACCA-3′; β-actin reverse: 5′- CTCCTTAATGTCACGCACGATTC-3′; P-gp forward: 5′-TTACACGTGGTTGGAAGC-3′; P-gp reverse: 5′-CATAGA TCAGCAGGAAAG-3′; HPRT forward: 5′-AAGAATGTTGTGATAAAAGGTGATGCT-3′; HPRT reverse: 5′-ACACATCCATGGGACTTCTGCCTC-3′. Samples were plated in qPCR optical plates and centrifuged at 2500 rpm for 1 min. qPCR was performed using an Applied Biosystems QuantStudio 7 Pro Real-Time PCR System (Thermo Fisher Scientific) consisting of a one-minute hold at 95 °C, followed by 40 cycles of 15 s at 95 °C and 30 s at 60 °C. Fold change was determined using ΔΔCt analysis.

### 4.7. Determination of P-Glycoprotein Expression via Western Blot

To determine relative P-gp expression, western blotting was performed. Following 24 h of transfection, cells were incubated with growth medium for an additional 72 h for a total post-transfection duration of 4 days. Cells were then washed with PBS and lifted using trypsin with 0.25% EDTA. After approximately 5 min of incubation at 37 °C and 5% CO_2_, trypsin was neutralized with an equal volume of growth medium, and cells were pelleted via centrifugation at 1000 rpm for 5 min at 4 °C. Following aspiration of the supernatant, cells were washed with ice-cold PBS and pelleted via centrifugation. The supernatant was removed, and RIPA buffer supplemented with protease inhibitors was added at an approximate concentration of 1 mL per 8 × 10^6^ cells. Cell solutions were kept on ice and vortexed every 5 min for a total of 30 min to acquire cell lysate. Following 2 min of centrifugation at 16,000× *g*, the supernatant was removed, and the pellet was discarded. The protein concentration of cell extracts was quantified using a BCA assay (Thermo Fisher Scientific Pierce BCA Assay Kit).

Protein extracts (10 µg) were combined with NuPAGE sample buffer and reducing agent and boiled at 100 °C for 5 min. Following brief centrifugation, samples were loaded into a NuPAGE 10% Bis-Tris gel and run in MOPS buffer at 200 V for 20–30 min. Proteins were transferred to a PVDF membrane at 20 V for 1 h in transfer buffer (3.04 g Tris-base, 14.4 g glycine, 100 mL methanol, 800 mL ultrapure water). Following transfer, the membrane was washed in TBS-T for 5 min (3x) and blocked for 30 min in a 5% nonfat milk solution prepared in TBS-T. The membrane was then divided horizontally at the 75 kDa mark and incubated overnight at 4 °C with gentle agitation in appropriate primary antibody prepared in 5% nonfat milk in TBS-T (1:5000 anti-P-gp 1° antibody for section greater than 75 kDa; 1:8000 anti-α tubulin 1° antibody for section less than 75 kDa). Following incubation with primary antibodies, membranes were washed in TBS-T for 5 min (3x). Membranes were then incubated with a 1:5000 dilution of anti-mouse horseradish peroxidase secondary antibody prepared in 5% nonfat milk in TBS-T at room temperature for 1 h. After secondary antibody incubation, membranes were washed in TBS-T for 5 min each (3x). Finally, membranes were treated with 1 mL of a 1:1 solution of Clarity Western ECL substrate for 1 min and immediately imaged on a ChemiDoc chemiluminescence system (Bio-Rad, USA). Densitometry analysis of protein bands was performed using ImageJ 1.54f software. Normalization factors for each lane were calculated relative to the intensity of the strongest α-tubulin signal, and P-gp expression was measured as a percentage of control samples.

### 4.8. Measurement of P-Glycoprotein Surface Concentration

To determine surface concentration of P-gp, flow cytometry experiments were performed. Following 24 h of transfection, cells were incubated with growth medium for an additional 72 h for a total post-transfection duration of 4 days. Cells were then washed with PBS and lifted using trypsin with 0.25% EDTA. After approximately 5 min of incubation at 37 °C and 5% CO_2_, trypsin was neutralized with an equal volume of growth medium, and cells were pelleted via centrifugation at 1000× *g* rpm for 5 min at 4 °C. Following aspiration of the supernatant, cells were resuspended in appropriate growth medium, stained with trypan blue, and counted using a Nexcelom Cellometer Auto 2000 Cell Viability Counter (Nexcelom, USA). Cells were plated at a density of 100,000–150,000 cells/well in a volume of 200 µL per well in a V-bottom plate. Following centrifugation at 1500 rpm at 4 °C for 5 min, the supernatant was discarded, and cells were washed in 200 µL of ice-cold PBS. Cells were then incubated on ice for 20 min in 100 µL of eFlour 780 fixable viability dye diluted in FACS buffer (2% fetal bovine serum in PBS) at a concentration of 1 µL per 1 mL. Following centrifugation, cells were washed with 200 µL of FACS buffer, and the supernatant was discarded. To stain for P-glycoprotein, cells were incubated on ice for 20 min with saturating concentrations of an Alexa Fluor 647-labeled anti-P-glycoprotein antibody. Cells were pelleted and washed in 200 µL of FACS buffer. Cells were then fixed in 100 µL of 2% paraformaldehyde solution in PBS and incubated for 20 min at room temperature. Following centrifugation, cells were washed with 200 µL of FACS buffer. The supernatant was discarded, and cells were resuspended in 200 µL FACS buffer.

Cells were analyzed using an Attune NxT flow cytometer (Thermo Fisher Scientific, USA). Alexa Fluor 647 (670/14 bandpass emission filter) and eFluor 780 (780/60 bandpass emission filter) were excited using a 638 nm laser, and instrument compensation was performed to minimize fluorescent spillover between channels. Data were collected in triplicate for each condition, and results were analyzed using the FlowJo 10.10.0 software (BD Biosciences, San Jose, CA, USA). To generate a standard curve for Alexa Fluor 647 fluorescence, Quantum Alexa Fluor 647 MESF (Molecules of Equivalent Soluble Fluorochrome) beads (Bangs Laboratories) were analyzed using the same PMT and compensation settings as the cell samples, and the QuickCal 3.0 software (Bangs Laboratories, Fishers, IN, USA) was used to correlate fluorescence intensity to Alexa Fluor 647 concentration. The fluorochrome-to-protein ratio of the fluorescently labeled antibody was measured using a NanoDrop One UV-Vis Spectrophotometer (Thermo Fisher Scientific, USA). To determine the density of P-glycoprotein per cell, the average MESF of each cell population calculated using the QuickCal 3.0 software was divided by the antibody F:P ratio.

### 4.9. Determination of Sensitivity of Transfected Cells to Chemotherapeutics

To determine the impact of siRNA-mediated changes in P-glycoprotein expression on drug sensitivity, cells were seeded in 96-well plates at appropriate densities (Table S1) and transfected for 24 h according to the method detailed above using sequence hs.Ri.ABCB1.13.1. Following transfection, cells were rinsed with PBS, and appropriate culture media containing varying concentrations of doxorubicin hydrochloride, paclitaxel, or vinblastine sulfate were added (*n* = 6 per condition). Plates were incubated for an additional 72 h at 37 °C and 5% CO_2_ for a total post-transfection duration of 4 days. Supernatants were aspirated to remove the drug, and cells were rinsed with PBS. To each well, 90 µL of appropriate media was added, followed by 10 µL of MTS reagent (Promega, USA). Cells were incubated at 37 °C for 1–4 h or until sufficient color change was observed. Plates were read at 490 nm on a BioTek Synergy HT microplate reader (Agilent Technologies, Santa Clara, CA, USA). The absorbance of the blank sample was subtracted from all samples, and viability data were normalized to that of cells treated with culture medium. IC_50_ values for each condition were determined using a four-parameter nonlinear regression model with variable slope in GraphPad Prism 10.2.0.

### 4.10. Characterization of Cell Interactions with Calcein-AM

To evaluate the impact of P-gp expression on substrate accumulation over short time scales, cells were seeded in black 96-well plates at appropriate densities and transfected for 24 h according to the protocol detailed above using sequence hs.Ri.ABCB1.13.1. Following transfection, cells were rinsed with PBS, and appropriate culture media without antibiotics was added for an additional 72 h for a total post-transfection duration of 4 days. Cells were then rinsed with PBS, and 50 µL of supplement-free culture media was added. A solution of calcein-AM was prepared in supplement-free culture media (0.5 µM for MDA435/LCC6 cells, 7.5 µM for MES-SA cells), and 50 µL were added to each well. Fluorescent measurements were immediately taken on a SpectraMax Gemini EM fluorescent plate reader (Molecular Devices, USA) 494/517 nm excitation/emission, at 37 °C every 30 s for one hour. Cells were then washed with PBS, and 50 µL of RIPA buffer was added to each well. Cells were incubated on ice with constant mixing for 30 min to obtain lysates. The fluorescence of cell lysates was then measured, and the protein concentration in each well was determined using a BCA assay (Thermo Scientific Pierce BCA Assay Kit). Fluorescent standard curves of cleaved calcein were prepared in supplement-free culture medium and in RIPA buffer to determine calcein concentration at each kinetic time point and in endpoint lysates, and calcein accumulation in cells was reported as pmol of calcein per µg of protein. Kinetic curves were plotted, and the slopes of the linear portions of each curve were determined using simple linear regression in GraphPad Prism 10.2.0 to estimate calcein-AM influx rates. Calcein influx rates and endpoint accumulation were log-transformed and plotted against P-glycoprotein expression for each cell line, and simple linear regression was performed using GraphPad Prism 10.2.0.

To visualize calcein accumulation in transfected cells, microscopy studies were performed. Cells were seeded in 24-well plates as detailed above. Following transfection, cells were rinsed with PBS and treated with calcein-AM (0.5 µM for MDA435/LCC6 cells, 7.5 µM for MES-SA cells) in supplement-free culture medium. Because saturating conditions of calcein-AM were desired, different concentrations of calcein-AM were used for each cell line due to the significantly higher native P-gp expression of MES-SA cells. Cells were incubated for 30 min at 37 °C and 5% CO_2_. Following two rinses with PBS, cells were fixed with 4% paraformaldehyde in PBS for 10 min at room temperature. Cells were rinsed two additional times with PBS. To each well, 500 µL PBS was added, and cells were immediately imaged on a BZ-X810 fluorescent microscope (Keyence, USA) equipped with a GFP filter. For each cell type, the fluorescent exposure settings were kept constant to enable qualitative comparisons across treatment groups.

### 4.11. Determination of Michaelis–Menten Characteristics of MES-SA Cells

To further characterize the kinetic behavior of the MES-SA cell line, a kinetics experiment was performed with varying concentrations of calcein-AM to evaluate the Michaelis–Menten parameters for each population of cells as a function of P-glycoprotein expression. Cells were transfected and treated with calcein-AM as detailed in the preceding sections using an initial calcein-AM concentration ranging from 62.5 to 10,000 nM. Kinetics measurements were taken over a period of 1 h, and cell lysates were obtained and measured as detailed above. Calcein fluorescence was plotted as a function of time for each substrate concentration, and the slope of the linear portion of the curve was calculated to determine the initial influx rate. Influx rates were plotted as a function of initial calcein-AM concentration to produce Michaelis–Menten curves for each cell population. Michaelis–Menten parameters were calculated using a nonlinear regression model in GraphPad Prism 10.2.0. To evaluate the relationship between Michaelis–Menten parameters and P-glycoprotein surface density, simple linear regression was performed on both untransformed data and log-transformed data to determine the model that best described the data, using R^2^ as a measure of goodness of fit.

### 4.12. Statistical Analysis

Statistical analyses were performed using GraphPad Prism 10.2.0. (GraphPad, Boston, MA, USA). Kinetic curves, growth rates, membrane fluidities, western blot quantification of P-gp expression, and flow cytometry measurements of P-gp surface density are displayed as mean ± standard deviation. All other data are presented as mean ± SEM. Growth rates were determined using an exponential growth model with log(population). IC_50_ values were determined using nonlinear regression of log(inhibitor) vs. response using a four-parameter model with variable slope. IC_50_ vs. P-gp, log(calcein accumulation) vs. P-gp, calcein influx rates, log(calcein influx rate) vs. P-gp, and Michaelis–Menten parameters vs. P-gp were modeled using simple linear regression. Michaelis–Menten parameters were determined using a nonlinear regression model. Statistical comparisons for each noted experiment were performed as follows: growth rate, one-way ANOVA; membrane fluidity, one-way ANOVA; sequence comparison of siRNA knockdown measured using qPCR, one-way ANOVA; and western blot vs. flow cytometry quantification of P-gp expression, multiple Mann–Whitney tests.

## 5. Conclusions

In this work, we show that the relationship between drug sensitivity and P-glycoprotein expression in vitro is governed by a linear correlation, suggesting that P-glycoprotein content can be used as a primary predictor of drug response. These results were shown in both a retrovirally transfected cell line and a drug-selected cell line, indicating that this relationship holds despite the origin of P-gp expression. While quantitative P-gp expression cannot be broadly used to predict IC_50_ values in isolation due to the variable inherent permeabilities of different cell types to substrates, the model established herein can be used to estimate drug sensitivity within a given biological system with defined boundary conditions. Additionally, we demonstrate that P-glycoprotein expression drives accumulation of the fluorescent substrate calcein-AM over short timescales, exhibiting first-order behavior. Future work will extend these results to an in vivo model, strengthening the therapeutic implications of these findings as a model to predict drug sensitivity of malignancies that overexpress P-glycoprotein.

## Figures and Tables

**Table 1:** Model parameters of linear relationships between IC_50_ and P-glycoprotein expression. Error terms represent the standard error of the mean calculated from the simple linear regression model.

Cell Type	Substrate	Slope ( $\frac{nM}{P\text{-}glycoprotein}$ )	Intercept (nM)	R^2^
MDA435/ LCC6	Doxorubicin	0.1387 ± 0.02747	−98.86 ± 244.0	0.8095
	Paclitaxel	9.472 × 10^−3^ ± 7.439 × 10^−4^	9.559 ± 6.609	0.9643
	Vinblastine	3.515 × 10^−4^ ± 2.966 × 10^−5^	0.2985 ± 0.2636	0.9590
MES-SA	Doxorubicin	0.1137 ± 0.05382	−341.9 ± 2487	0.4267
	Paclitaxel	4.074 × 10^−2^ ± 1.005 × 10^−2^	−387.1 ± 464.2	0.7372
	Vinblastine	1.061 × 10^−2^ ± 2.520 × 10^−3^	−64.47 ± 116.5	0.7470

**Table 2:** Model parameters of semi-log relationships of calcein accumulation and influx rate as a function of P-glycoprotein expression. Error terms represent standard error of the mean calculated from the simple linear regression model.

Cell Type	Parameter	Slope	Intercept (nM)	R^2^
MDA435/ LCC6	Calcein steady state accumulation	−3.435 × 10^−5^ ± 3.558 × 10^−6^	−0.7738 ± 0.03161	0.9395
	Calcein influx rate	−2.885 × 10^−5^ ± 3.667 × 10^−6^	−2.257 ± 0.03258	0.9166
MES-SA	Calcein steady state accumulation	−9.175 × 10^−6^ ± 1.781 × 10^−6^	−0.5066 ± 0.08230	0.8157
	Calcein influx rate	−1.095 × 10^−5^ ± 1.955 × 10^−6^	−1.056 ± 0.09036	0.8393

## Data Availability

The original contributions presented in this study are included in the article and Supplementary Materials. Further inquiries can be directed to the corresponding author. Any additional raw data will be made available by the authors upon request.

## Supplementary Materials

The following supporting information can be downloaded at: https://www.mdpi.com/article/10.3390/molecules30143014/s1, Figure S1: Characterization of MDA435/LCC6 and MES-SA cells; Figure S2: Validation of transfection and RNAi recruitment; Figure S3: Determination of optimal transfection duration for modulation of P-glycoprotein expression; Figure S4: Western blots of transfected MDA435/LCC6 cell lysates; Figure S5: Western blots of transfected MES-SA cell lysates; Figure S6: Flow cytometry gating scheme and antibody standard curve for MDA435/LCC6 cells; Figure S7: Visualization of flow cytometry gating for P-glycoprotein expression by transfected MDA435/LCC6 cells; Figure S8: Flow cytometry gating scheme and antibody standard curve for MES-SA cells; Figure S9: Visualization of flow cytometry gating for P-glycoprotein expression by transfected MES-SA cells; Figure S10: IC_50_ curves for transfected MDA435/LCC6 cells; Figure S11: IC_50_ curves for transfected MES-SA cells; Figure S12: Linear and semi-log models of the relationship between K_m_ and P-glycoprotein surface density in MES-SA cells; Table S1: Numerical P-glycoprotein surface expression of all cell populations; Table S2: Cell seeding densities used for siRNA transfection experiments.

## Abbreviations

The following abbreviations are used in this manuscript:

P-gp	P-glycoprotein
MDR	Multidrug resistance
IMEM	Improved minimum essential medium
PDA	Pyrene decanoic acid
