# Supplementary material for: A Quantitative Model of Chemotherapeutic Drug Sensitivity as a Function of P-Glycoprotein Expression
Source: Molecules. 2025 Jul 18;30(14):3014. doi: 10.3390/molecules30143014 (PMC12300152; doi:10.3390/molecules30143014)
Supplement: Supplementary file 1 [file molecules-30-03014-s001.zip › molecules-3685342-supplementary.pdf]

molecules

Supplementary Materials

A quantitative model of chemotherapeutic drug sensitivity as a function of P-glycoprotein expression

Cara M. Robertus [1], Nisha Kannan [1] and David Putnam [1,2,*]

[1] Meinig School of Biomedical Engineering, Cornell University, Ithaca, NY 14850, USA; cr546@cornell.edu (C.M.R.); nk489@cornell.edu (N.K.)
[2] Smith School of Chemical and Biomolecular Engineering, Cornell University, Ithaca, NY 14850, USA
* Correspondence: dap43@cornell.edu

Contents

Figures S1-S12

Tables S1-S2

 https://doi.org/10.3390/xxxxx

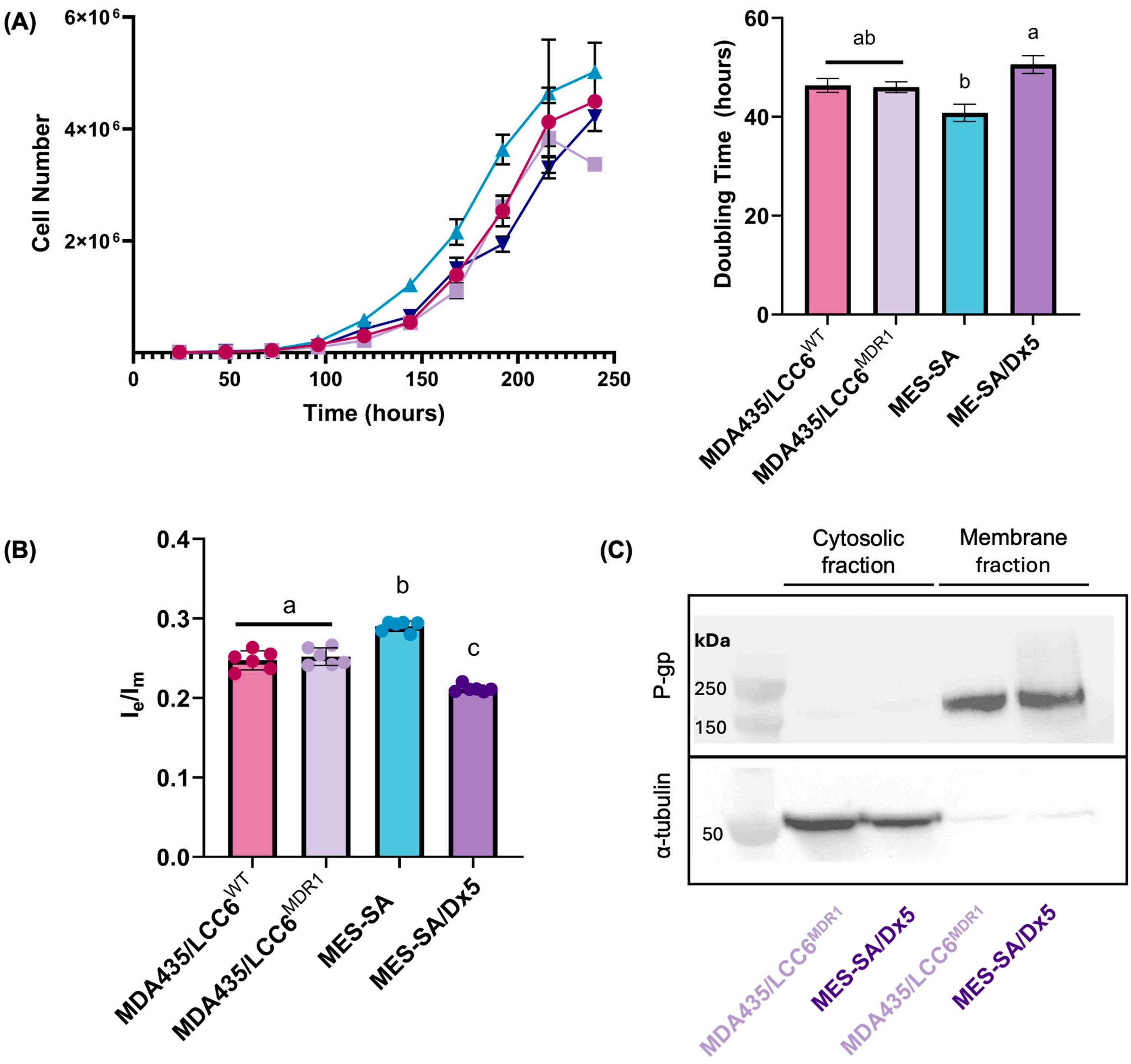

Figure S1. Characterization of MDA435/LCC6 and MES-SA cells. (A) Growth curves (left) and growth rates (right) of all cell lines. Error bars represent standard deviation, $n = 3$. Statistical significance of growth rates determined using a one-way ANOVA. Letters represent levels of statistical equivalence. (B) Membrane fluidity of all cell lines. An increasing ratio of excimer to monomer incorporation into the cell membrane (I_e/I_m) represents increased membrane fluidity. Error bars represent standard deviation, $n = 6$. Statistical significance was determined using a one-way ANOVA. Letters represent levels of statistical equivalence. (C) Representative western blot of cytosolic and membrane-associated subcellular protein fractions from MDA435/LCC6^{MDR1} cells and MES-SA/Dx5 cells.

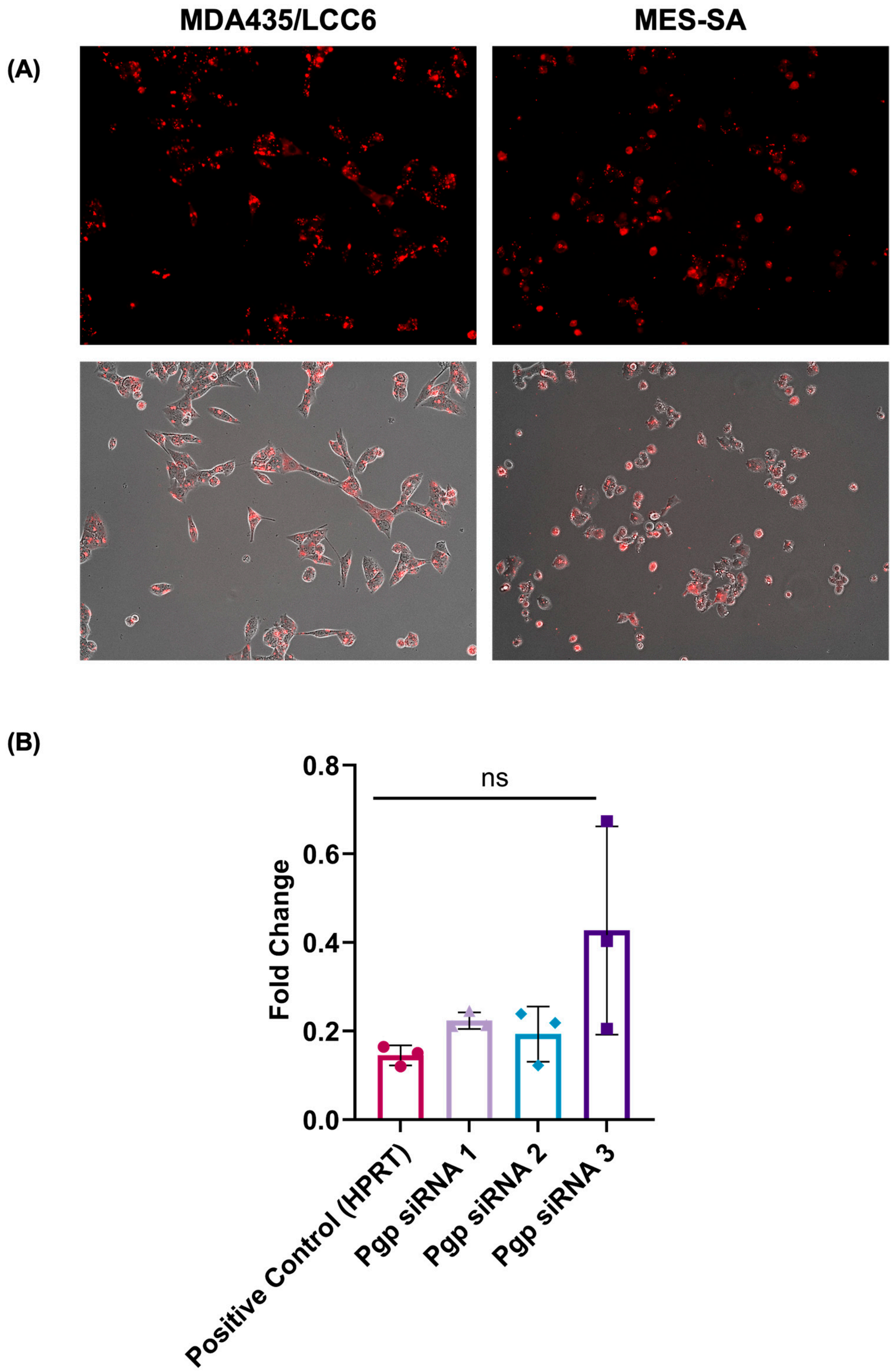

Figure S2. Validation of transfection and RNAi recruitment. (A) Representative fluorescence images of MDA435/LCC6 and MES-SA cells transfected with a TYE 563-labeled control siRNA sequence using Lipofectamine RNAiMAX. Fluorescent and brightfield overlay images convey high transfection efficiency. (B) Representative mRNA fold change data following a 24-hour transfection of MDA435/LCC6 cells with 20nM of various siRNA sequences. HPRT was used as a housekeeping gene. Statistical significance was determined using one-way ANOVA. Error bars represent standard deviation, $n = 3$. “Ns” represents “no significance,” indicating no statistical difference between groups.

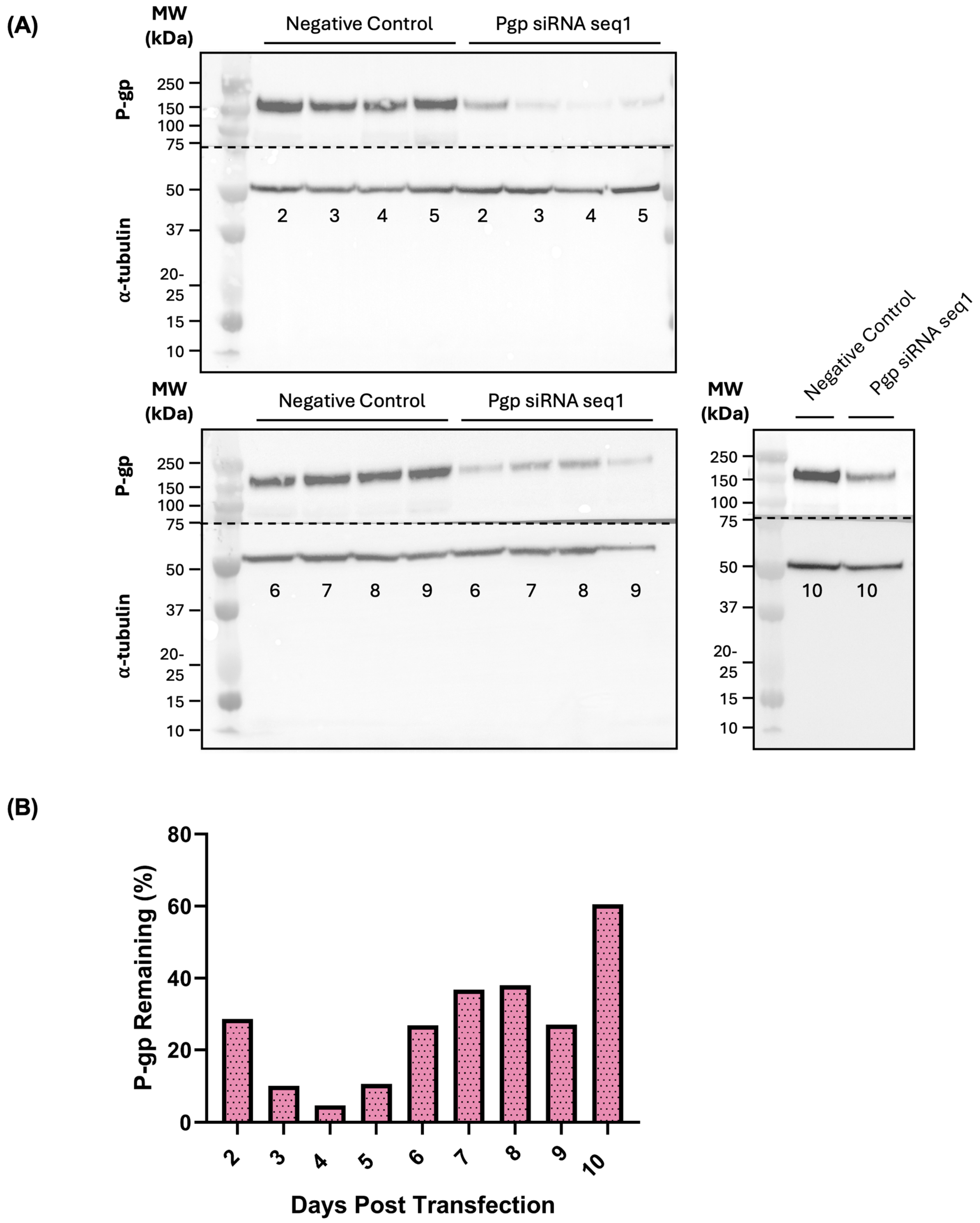

Figure S3. Determination of optimal transfection duration for modulation of P-glycoprotein expression. (A) Western blots of total protein lysates obtained from MDA435/LCC6^{MDR1} cells transfected for varying durations. Numbers indicate transfection duration in days. (B) Quantification of western blot data conveying relative P-glycoprotein expression as a function of transfection duration. One measurement was acquired per time point.

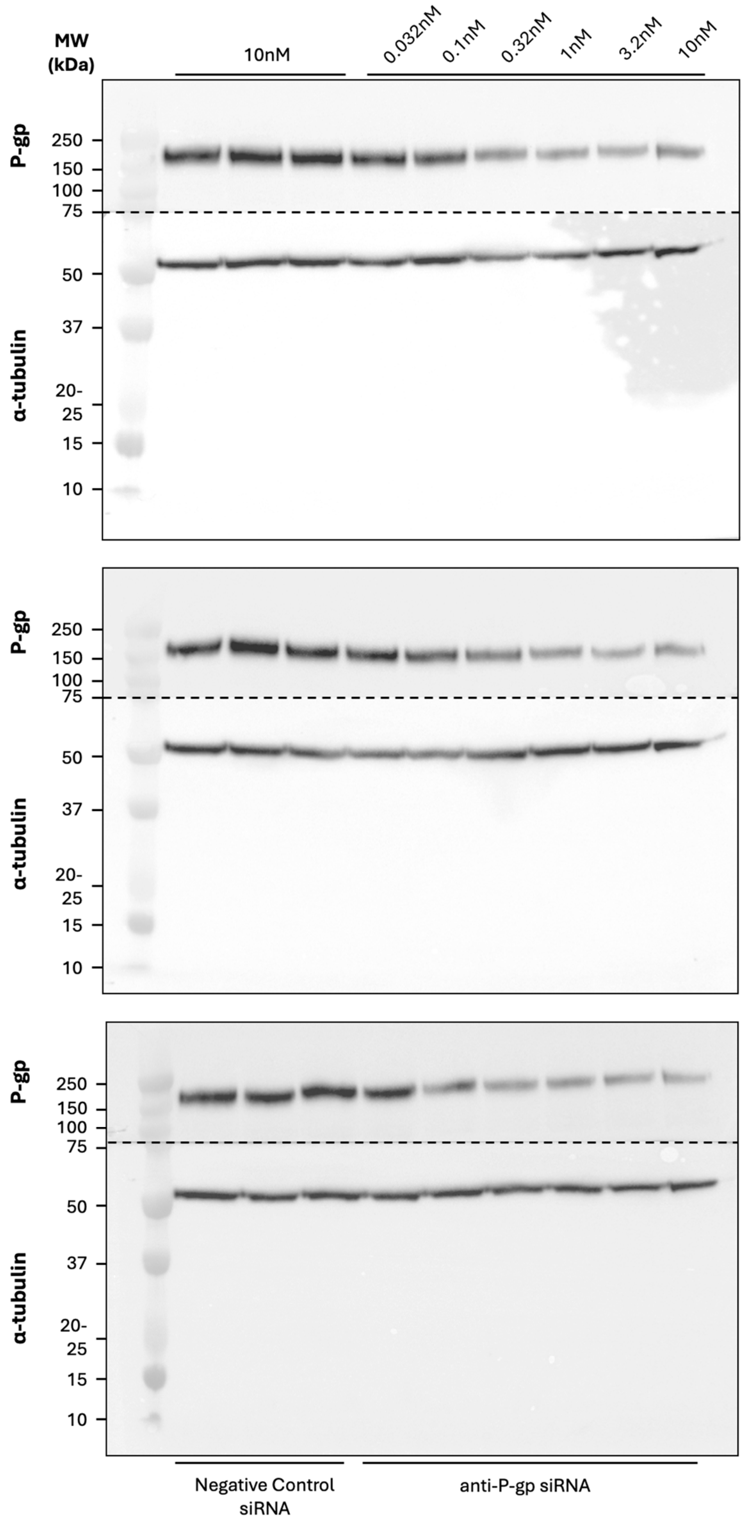

Figure S4. Western blots of transfected MDA435/LCC6 cell lysates. Western blots used to determine relative changes in P-glycoprotein expression following transfection with increasing concentrations of siRNA (n = 3). The negative control siRNA sequence was administered at a constant concentration of 10 nM. α-tubulin was used as a loading control, and densitometry analysis was used to quantify relative expression.

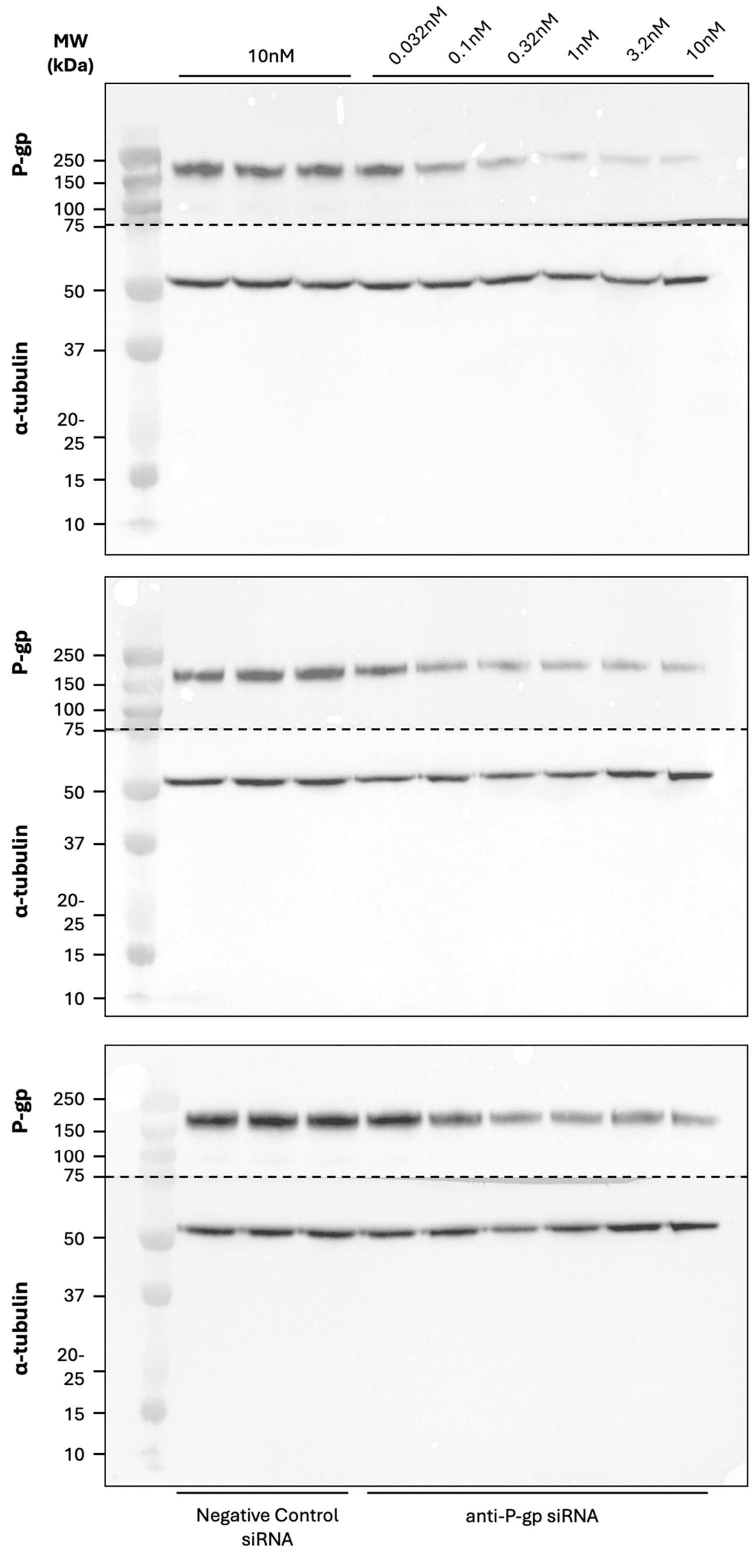

Figure S5. Western blots of transfected MES-SA cell lysates. Western blots used to determine relative changes in P-glycoprotein expression following transfection with increasing concentrations of siRNA ($n = 3$). The negative control siRNA sequence was administered at a constant concentration of 10 nM. α-tubulin was used as a loading control, and densitometry analysis was used to quantify relative expression.

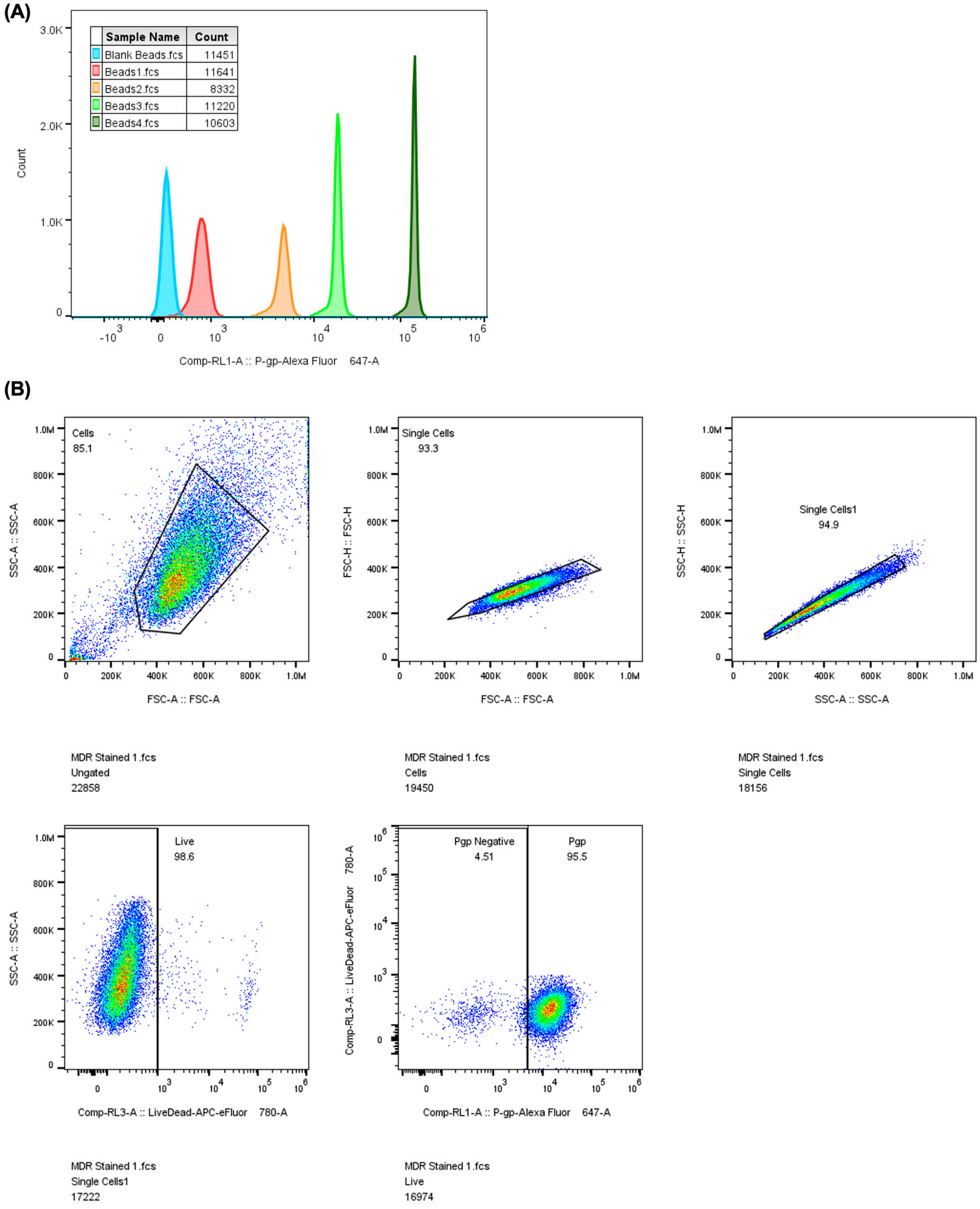

Figure S6. Flow cytometry gating scheme and antibody standard curve for MDA435/LCC6 cells. (A) Fluorescent standard curve of AlexaFluor647 signal using Bangs Laboratories bead standards. Standard curve values were used to calculate the number of antibodies bound to each cell at saturation. (B) Gating scheme used to identify live, single cells. This subpopulation was further gated for P-glycoprotein expression as measured by fluorescent intensity of an AlexaFluor 647-labeled antibody.

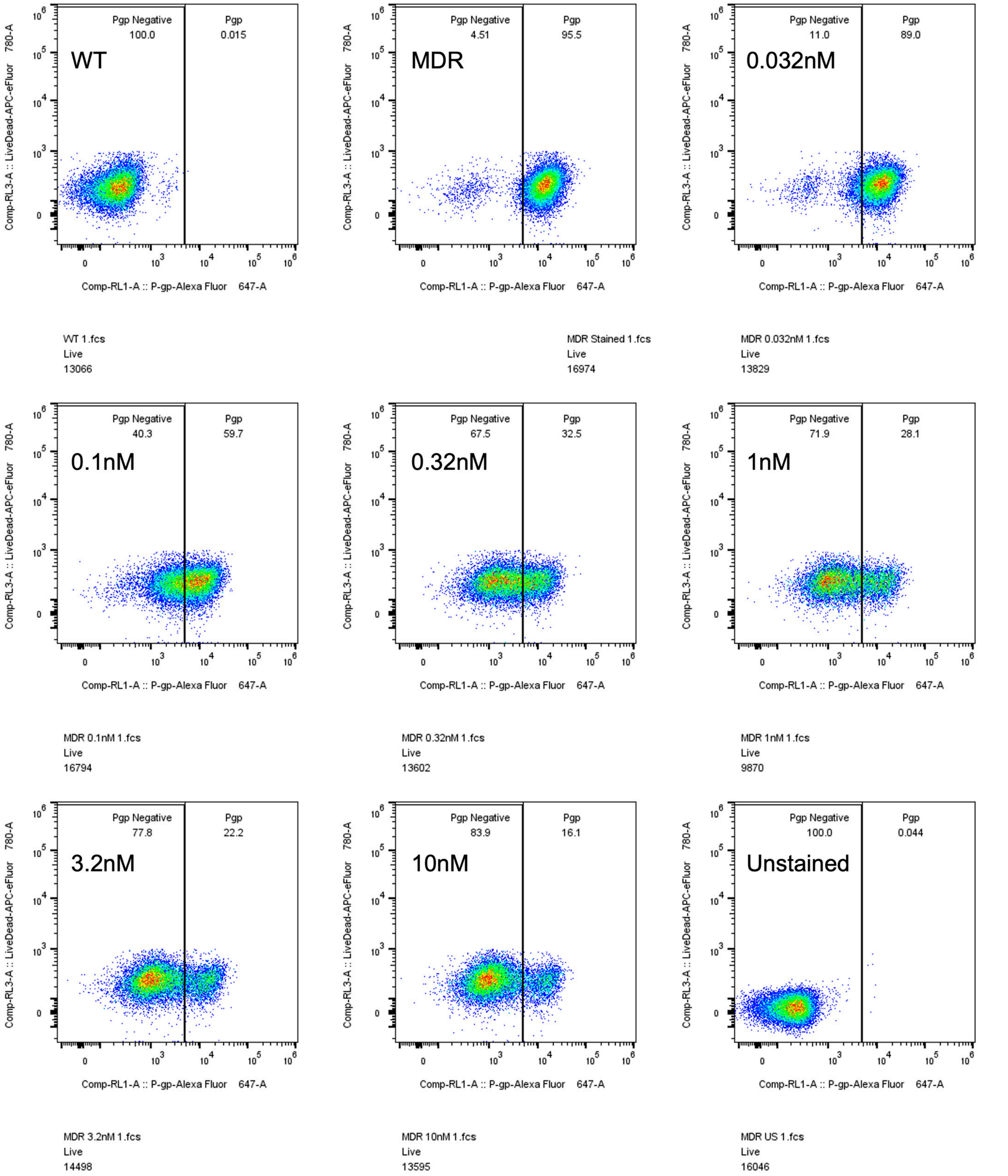

Figure S7. Visualization of flow cytometry gating for P-glycoprotein expression by transfected MDA435/LCC6 cells. Gating scheme used to quantify antibody binding to P-glycoprotein in a subpopulation of live, single cells subjected to transfection with varying concentrations of siRNA. Due to the bimodal population distribution evident at higher concentrations of siRNA, a weighted average of fluorescent intensity in two adjacent gates was used to determine the population average.

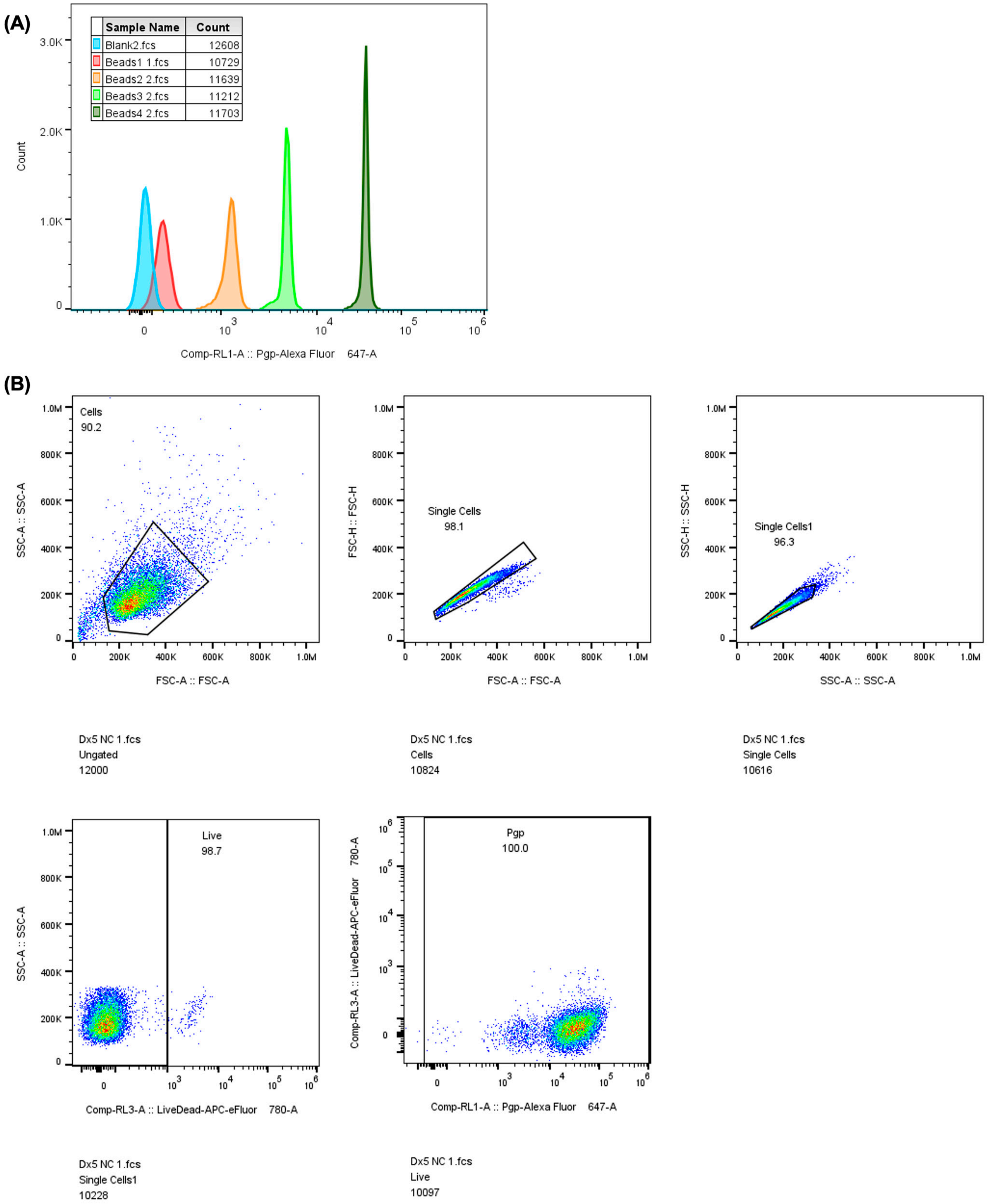

Figure S8. Flow cytometry gating scheme and antibody standard curve for MES-SA cells. (A) Fluorescent standard curve of AlexaFluor647 signal using Bangs Laboratories bead standards. Standard curve values were used to calculate the number of antibodies bound to each cell at saturation. (B) Gating scheme used to identify live, single cells. This subpopulation was further gated for P-glycoprotein expression as measured by fluorescent intensity of an AlexaFluor 647-labeled antibody.

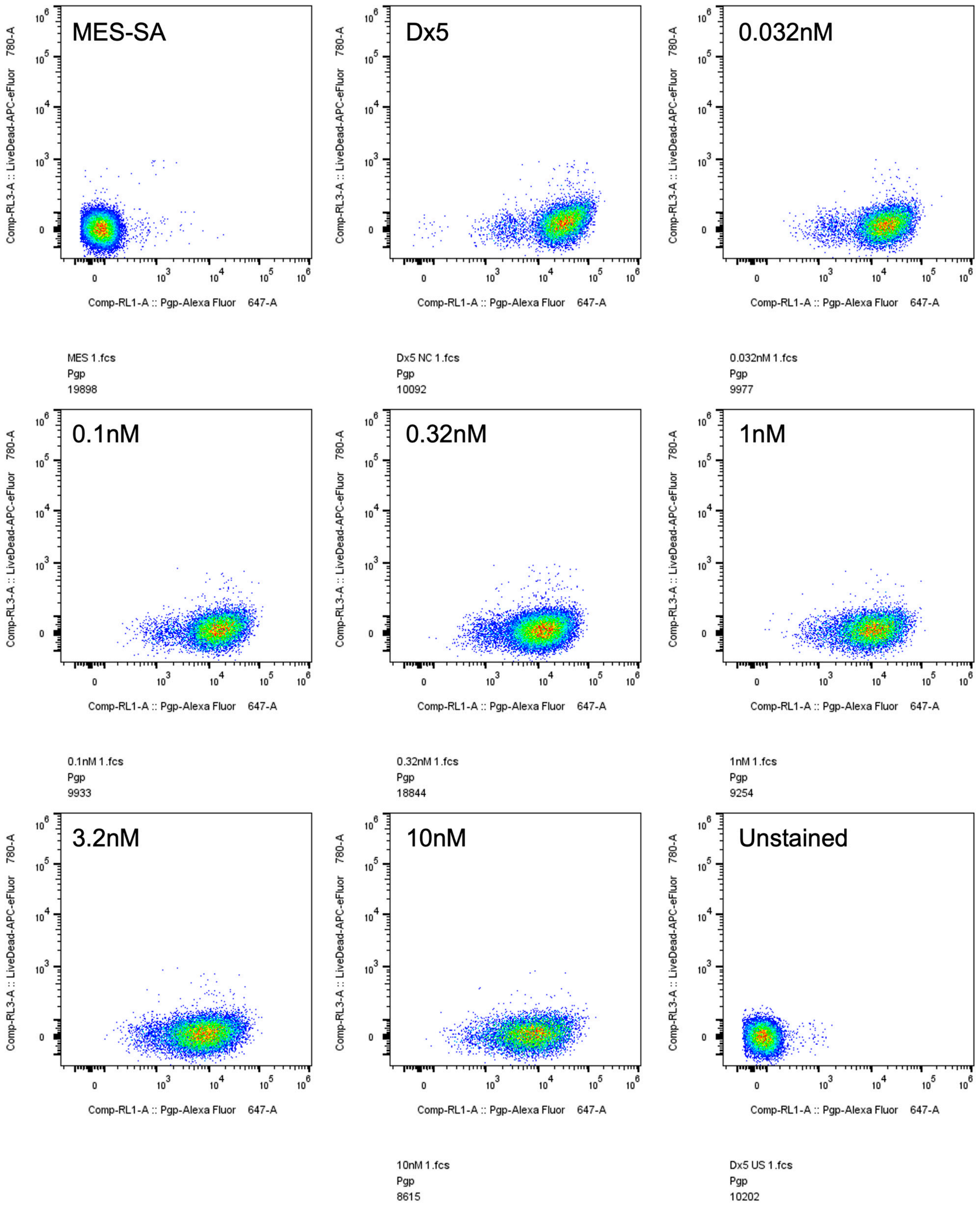

Figure S9. Visualization of flow cytometry gating for P-glycoprotein expression by transfected MES-SA cells. Gating scheme used to quantify antibody binding to P-glycoprotein in a subpopulation of live, single cells subjected to transfection with varying concentrations of siRNA. Because the population distribution remained fairly uniform across concentrations of siRNA, the average fluorescent intensity of the population was directly quantified in lieu of a population threshold.

(A)

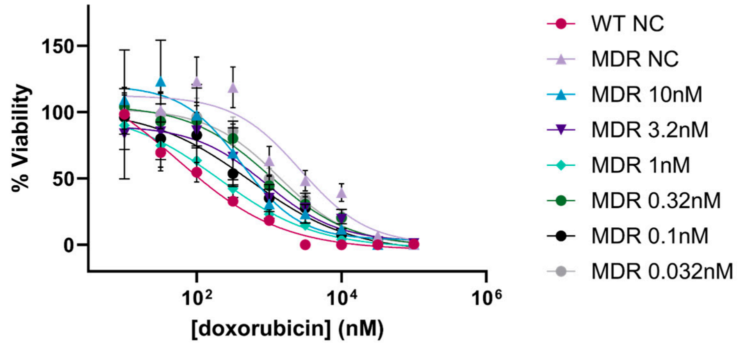

Sample	IC_{50} (nM)	SE IC_{50}	R^2
WT NC	52.81	32.17	0.9534
MDR NC	2716	772.49	0.8717
MDR 10nM	406.6	113.02	0.846
MDR 3.2nM	901.9	379.48	0.7598
MDR 1nM	203.7	43.37	0.9768
MDR 0.32nM	1017	239.60	0.9251
MDR 0.1nM	544.4	191.82	0.9092
MDR 0.032nM	1374	271.28	0.9345

(B)

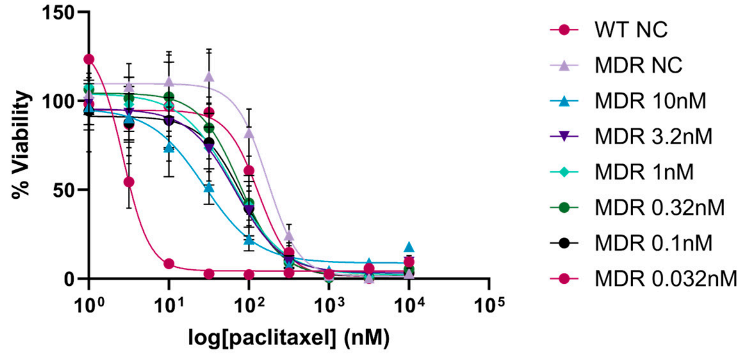

Sample	IC_{50} (nM)	SE IC_{50}	R^2
WT NC	2.673	0.44	0.9281
MDR NC	170.1	14.55	0.9605
MDR 10nM	27.58	4.24	0.9297
MDR 3.2nM	67.54	9.75	0.9248
MDR 1nM	63	9.76	0.9228
MDR 0.32nM	76.84	7.61	0.9561
MDR 0.1nM	83.37	9.14	0.947
MDR 0.032nM	132.2	24.54	0.8279

(C)

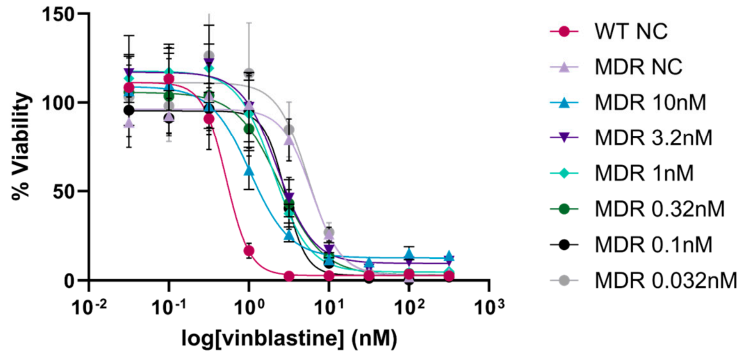

Sample	IC_{50} (nM)	SE IC_{50}	R^2
WT NC	0.5212	0.04	0.9668
MDR NC	6.117	0.64	0.9397
MDR 10nM	1.033	0.11	0.9403
MDR 3.2nM	2.246	0.28	0.9249
MDR 1nM	2.009	0.16	0.9663
MDR 0.32nM	2.407	0.32	0.9274
MDR 0.1nM	2.846	0.22	0.9448
MDR 0.032nM	5.502	0.84	0.8827

Figure S10. IC_{50} curves for transfected MDA435/LCC6 cells. IC_{50} curves and associated model parameters for transfected MDA435/LCC6 cells treated with (A) doxorubicin, (B) paclitaxel, and (C) vinblastine. Cell viability data was fit to a nonlinear regression model with variable slope to determine IC_{50} values. The model fit is reported as R^2. IC_{50} values and associated standard errors were used to generate linear models correlating drug sensitivity to P-glycoprotein expression. Error bars represent standard deviation, $n = 6$.

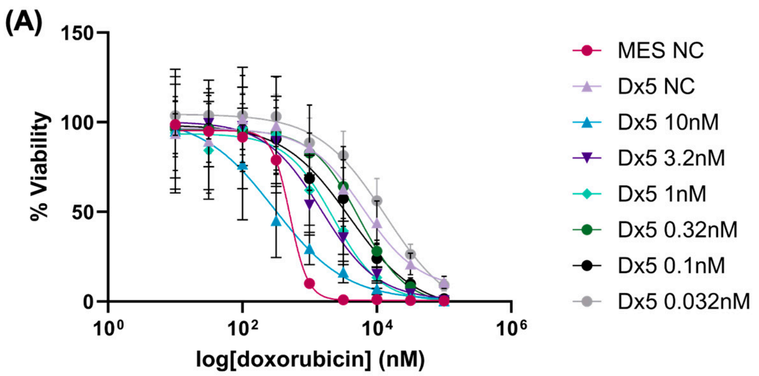

Sample	IC_{50} (nM)	SE IC_{50}	R^2
MES NC	510.4	20.57	0.9911
Dx5 NC	6245	2695.23	0.7478
Dx5 10nM	295.5	134.95	0.7977
Dx5 3.2nM	1512	411.24	0.8512
Dx5 1nM	2242	725.96	0.7609
Dx5 0.32nM	5369	1193.45	0.8804
Dx5 0.1nM	3944	1106.31	0.9008
Dx5 0.032nM	13466	5033.28	0.919

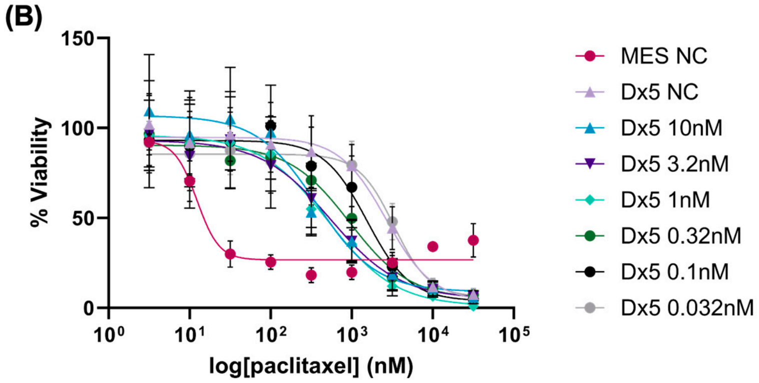

Sample	IC_{50} (nM)	SE IC_{50}	R^2
MES NC	12.32	1.24	0.8878
Dx5 NC	2669	517.86	0.8738
Dx5 10nM	369.5	102.37	0.8189
Dx5 3.2nM	552.1	197.33	0.7757
Dx5 1nM	510.2	130.42	0.8662
Dx5 0.32nM	938.1	243.48	0.8463
Dx5 0.1nM	1544	272.31	0.8815
Dx5 0.032nM	3319	438.59	0.9018

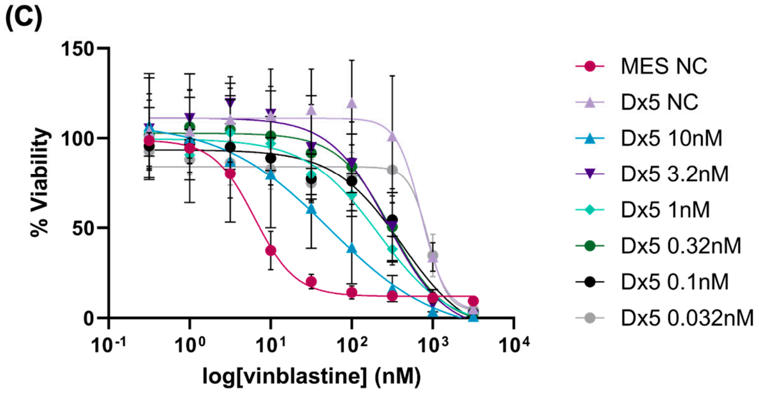

Sample	IC_{50} (nM)	SE IC_{50}	R^2
MES NC	6.329	0.52	0.9701
Dx5 NC	720.5	127.42	0.7708
Dx5 10nM	52.87	30.77	0.7587
Dx5 3.2nM	277.7	71.18	0.8671
Dx5 1nM	213.7	58.12	0.9018
Dx5 0.32nM	307.3	91.44	0.8024
Dx5 0.1nM	414.6	117.06	0.9148
Dx5 0.032nM	879.4	96.60	0.8454

Figure S11. IC_{50} curves for transfected MES-SA cells. IC_{50} curves and associated model parameters for transfected MES-SA cells treated with (A) doxorubicin, (B) paclitaxel, and (C) vinblastine. Cell viability data was fit to a nonlinear regression model with variable slope to determine IC_{50} values. The model fit is reported as R^2. IC_{50} values and associated standard errors were used to generate linear models correlating drug sensitivity to P-glycoprotein expression. Error bars represent standard deviation, *n* = 6.

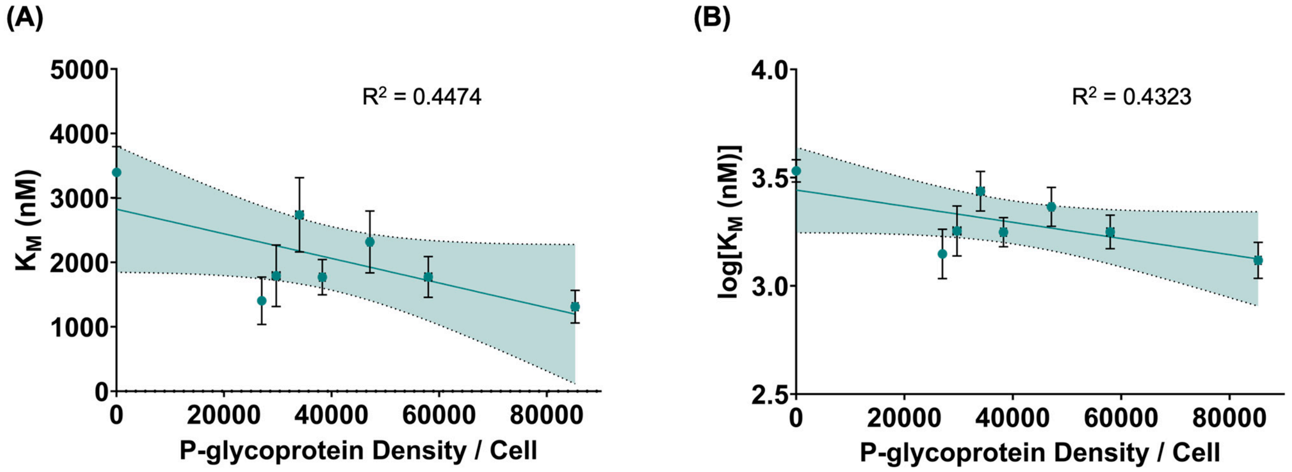

Figure S12. Linear and semi-log models of the relationship between K_m and P-glycoprotein surface density in MES-SA cells. (A) Linear model of K_m versus P-glycoprotein expression in transfected populations of MES-SA cells. (B) Linear model of log-transformed K_m versus P-glycoprotein expression in transfected populations of MES-SA cells. Neither model provides a robust correlation to the data, unlike the V_{max} parameter. Error bars represent the standard error of the mean, $n = 6$. Shaded areas represent the 95% confidence interval of the best-fit line.

Table S1. Numerical P-glycoprotein surface expression of all cell populations. Surface density of P-glycoprotein for all cell populations measured using flow cytometry, *n* = 6. "NC" refers to cell populations treated with non-targeting control siRNA. Standard error values are displayed to align with graphs shown in the main manuscript, in which standard error is used to correlate with error values calculated for IC_{50} model parameters.

Population	P-gp Surface Density	Standard Error
**MDA435/LCC6**		
WT	10.572	8.580
MDR NC	17,395.200	309.618
MDR 10nM	3499.474	199.716
MDR 3.2nM	4632.248	81.100
MDR 1nM	5354.811	75.985
MDR 0.32nM	5661.316	52.899
MDR 0.1nM	8316.980	412.844
MDR 0.032nM	12,859.650	329.286
MES-SA		
MES	28.891	19.811
Dx5 NC	85,269.858	533.300
Dx5 10nM	27,003.496	146.463
Dx5 3.2nM	29,687.539	702.938
Dx5 1nM	34,014.548	674.981
Dx5 0.32nM	38,278.476	515.203
Dx5 0.1nM	47,118.155	388.227
Dx5 0.032nM	57,974.319	625.062

Table S2. Cell seeding densities used for siRNA transfection experiments. Variable seeding densities for MES-SA and MES-SA/Dx5 cells were used to compensate for increased cytotoxicity of the transfection complex observed in MES-SA/Dx5 cells.

Cell Type	96-well seeding density	24-well seeding density	6-well seeding density
MDA435/LCC6	2,500	8,000	40,000
MDA435/LCC6^{MDR1}	2,500	8,000	40,000
MES-SA	5,000	10,000	100,000
MES-SA/DX5	13,500	40,000	250,000
